# Enhanced physiological performance and induced genetic variation in radish (*Raphanus sativus* L.) under gamma irradiation via silver chromate/aluminum-organic framework application

**DOI:** 10.1186/s12870-026-08715-3

**Published:** 2026-04-25

**Authors:** Hend Mostafa Habib, Marwa Mahmoud El-Attar, Reda M Abdelhameed

**Affiliations:** 1 Radioisotopes Department, Nuclear Research Center, Egyptian Atomic Energy Authority, Cairo, 11787 Egypt; 2 Applied Organic Chemistry Department, Chemical Industries Research Institute, National Research Centre, Giza, 12622 Egypt

**Keywords:** *Raphanus sativus*, Gamma Irradiation, Metal–Organic Frameworks (MOFs), SCoT markers, Al-MOF, Plant growth promotion, Genetic variation, Secondary metabolites

## Abstract

This study investigates the unconventional effects of gamma irradiation and/or metal-organic frameworks (MOFs) on *Raphanus sativus*. Silver chromate/aluminum-based MOFs were applied to both irradiated and non-irradiated *R. sativus* seeds. The evaluation was based on physiological characteristics—including phenotypic measurements and the content of ascorbic acid and total phenols—alongside genetic variation analysis using Start Codon Targeted (SCoT) markers.

Improved growth parameters, including shoot height, root length, leaves number, and leaf area, were observed in the Al-MOF, Ag_2_CrO_4_/Al-MOF, and/or irradiation treatments. Notably, at 9 days after spray (DAS), plants from irradiated seeds and those treated with Ag_2_CrO_4_/Al-MOF exhibited enhanced phenotypic development (four leaves) compared to non-irradiated controls with noticeable, varying changes in leaf area. Conversely, chlorophyll content was not significantly affected. Furthermore, all treatments led to a significant increase in ascorbic acid and total phenol content relative to the control.

By the conclusion of the experiment, MOFs perform effectively in conjunction with gamma irradiation (10 Gy) synergistically to enhance *R. sativus* features. Genetic analysis via SCoT revealed that MOFs in conjunction with gamma irradiation induced DNA variations. Specifically, when irradiation accompanied by Ag_2_CrO_4_/Al-MOF resulted in six negative unique markers, leading to genetic variants and instability relative to the control. These SCoT results remain complex and warrant further investigation. These findings promote the application of MOF products alongside irradiation in agriculture to optimize resource use. However, further research is required, particularly regarding the long-term effects of Al^3+^-MOFs.

**Supplementary Information:**

The online version contains supplementary material available at 10.1186/s12870-026-08715-3.

## Introduction

In the search for sustainable functional foods, the leaves of tuberous root crops have emerged as potent sources of bioactive compounds, often surpassing the nutritional density of the roots themselves. Radish (*Raphanus sativus* L.), a prominent member of the Brassicaceae family, is particularly notable for its high polyphenolic content and antioxidant capacity. These attributes make radish leaves a prime candidate for the development of supplements aimed at mitigating metabolic diseases and improving gut health. However, optimizing the concentration of these secondary metabolites and ensuring robust plant growth requires innovative physiological and molecular priming strategies [1, 2].

One established method for stimulating plant metabolism is gamma irradiation. While high doses (100–300 Gy) are known to disrupt protein synthesis and gas exchange [3], low-dose irradiation—a phenomenon known as hormesis—can act as a powerful eustressor. Doses ranging from 2.5 to 50 Gy have been shown to significantly elevate the accumulation of flavonoids and phenolics, while simultaneously improving germination and biomass in various vegetable crops [4]. Despite these benefits, the physical application of such treatments often lacks a delivery mechanism that can provide sustained or targeted support to the plant’s nutritional pathways [5–10].

Parallel to radiological advancements, nanotechnology has revolutionized agricultural inputs through the foliar application of nanoparticles (NPs). These innovations are highly effective when used as nutritional supplements, nano-fertilizers, herbicides, post-harvest preservatives, or nanosensors. Plants generally absorb foliar-applied nanoparticles through various entry points, including the stomata, stigma, wounds, cracks, hydathodes (water pores), trichomes, endocytosis, ion channels, and protein carriers. Among these, the primary pathway for leaf surface absorption is thought to be stomatal penetration. Other mechanisms, such as epidermal adsorption and internalization, also contribute to metal uptake, although they are often limited by the small size of the pores. Typically, young, immature leaves exhibit a higher absorption capacity than mature leaves. This is because immature leaves possess thinner epicuticular wax layers and are physiologically less developed, presenting fewer barriers to metal penetration [11]. Foliar-applied nanofertilizers offer several advantages over traditional soil-applied fertilizers, including rapid absorption and greater cost-effectiveness with minimal impact on soil health. These nanofertilizers can efficiently deliver essential vitamins or trace elements that may be deficient in the soil, quickly alleviating nutrient scarcity in plants. Furthermore, the slow-release mechanism of nanofertilizers enhances the uptake efficiency of primary macronutrients, such as nitrogen (N), phosphorus (P), and potassium (K).

To maximize this efficiency, Metal-Organic Frameworks (MOFs) have recently gained attention. These porous, crystalline materials—composed of metal nodes and organic linkers—offer an unparalleled surface area for the encapsulated, controlled release of nutrients or bioactive triggers. MOFs hold immense potential due to their ability to encapsulate a variety of molecules within their wide pore-size distributions. They can absorb specific nutrients, fertilizers, pesticides, or insecticides and release them at targeted sites, functioning as smart delivery systems. This capability allows for the fabrication of controlled-release mechanisms that enhance crop growth and productivity [12]. Furthermore, these porous compounds are frequently engineered to provide precise control over internal pore dimensions and physical properties by varying the combination of metal cores and organic linkers [13–16]. While trivalent ions like Al^3+^ and Cr^+ 3^ are known to influence root development and chlorophyll content [17–19], their behavior when integrated into a MOF architecture (Al-MOF or Ag_2_CrO_4_/Al-MOF) remains an emerging frontier in plant science.

The integration of these two distinct priming agents—low-dose gamma irradiation and Al/Cr-based MOFs—presents a novel synergistic opportunity. However, enhancing the physiological and nutraceutical profile of a crop also necessitates a rigorous assessment of genetic stability [20]. The Start Codon Targeted (SCoT) marker technique offers a robust, reproducible means of monitoring these genomic responses. By targeting conserved regions flanking the ATG start codon, SCoT markers can detect structural DNA alterations—such as deletions or strand breaks—that may arise from radiological or chemical treatments, even in species without a reference genome [21–24].

Despite the documented individual benefits of gamma irradiation and nanotechnology, their combined and potentially synergistic impact on the physiological, biochemical, and genetic landscapes of *Raphanus sativus* remains largely unexplored. Therefore, this study hypothesized that low-dose gamma irradiation (10 Gy) and foliar application of Al-MOF and Ag_2_CrO_4_/Al-MOF would individually enhance the growth and bioactive compound content of *Raphanus* sativus. Further, this study hypothesized that the combined application of irradiation and MOFs would result in a synergistic effect, leading to greater improvements than either treatment alone. Finally, this study hypothesized that these treatments would induce measurable genetic variations, detectable by SCoT markers, and that these variations might correlate with the observed phenotypic changes.

## Materials and methods

### Chemicals

Aluminum chloride (AlCl_3_, 99.5%) and benznedicarboxylic acid (H_2_BDC, 99%) were purchased from Merck Inc. Nitric acid (HNO_3_), sulfuric acid (H_2_SO_4_), perchloric acid (HClO_4_), ammonium molybdate, oxalic acid, EDTA, metaphosphoric acid, acetic acid, sodium carbonate, ethanol, methanol, dimethyl formamide (DMF) and acetonitrile were supplied from Sigma–Aldrich.

### Seed selection

Seeds of radish (*Raphanus sativus* cv. Balady) were obtained from a licensed Egyptian supplier. To ensure experimental uniformity, seeds of equal size and consistent color were selected to achieve the recommended planting density. These seeds were then subjected to low-dose gamma irradiation (0 and 10 Gy) using a ^60^Co source (Indian Gamma Cell) at the National Center for Radiation Research and Technology (NCRRT), Nasr City, Cairo, Egypt. The irradiation was conducted at a dose rate of 0.79670 kGy/h.

### Synthesis of Al-MOF

The Al-MOF was synthesized using the following solvothermal procedure. A mixture of AlCl_3_ (0.266 g, 2 mmol) and terephthalic acid (H_2_BDC, 0.498 g, 3 mmol) was combined with 80 mL of N, N-dimethylformamide (DMF) and 20 mL of methanol in a Teflon-lined autoclave. The resulting slurry was heated to 150 °C for 20 h. After cooling to room temperature, the product was collected via centrifugation. To remove residual DMF from the pores, the solid was washed repeatedly with 100 mL of methanol and 100 mL of acetone. Finally, the resulting Al-MOF (0.312 g) was dried in a vacuum oven at 100 °C for 24 h and stored at room temperature until further use.

### Fabrication of Ag_2_CrO_4_/Al-MOF

The Ag_2_CrO_4_/Al-MOFcomposite was synthesized via the precipitation method. Initially, 0.2 g of Al-MOF was dispersed in 25 mL of absolute ethanol and sonicated (or stirred) for 30 min. Subsequently, 78 mg of Na_2_CrO_4_ and 10 mg of AgNO_3_ were added to the dispersion. The reaction mixture was then agitated at room temperature for six hours. The resulting Ag2CrO_4_/Al-MOF was collected, washed repeatedly to remove impurities, and dried under vacuum at 60 °C.

### Characterizations

The crystalline structure and phase purity of the samples were examined via X-ray diffraction (XRD) using an X’pert Pro-Panalytical (Netherlands) instrument with CuK\alpha radiation, operating at 40 kV and 40 mA. XRD patterns were recorded over a 2 theta range of 3° to 80° with a step size of 0.05°. Furthermore, the surface morphology of Ag_2_CrO_4_, Al-MOF, and the Ag_2_CrO_4_/Al-MOF composite was analyzed using field emission-scanning electron microscopy (FE-SEM) (QUANTA FEG250, Czech Republic) at an accelerating voltage of approximately 20 kV.

### Experimental design and location

The seeds were thoroughly rinsed with distilled water, surface-sterilized in 1% (v/v) sodium hypochlorite for approximately 2 min, and then air-dried at room temperature (21 °C) for one hour. The experiment was conducted during the 2021 winter season (commencing after December 21) under a light/dark photoperiod of 11/13 h at 21 °C. The study was carried out in a greenhouse without supplemental lighting at the experimental area of the Radioisotopes Department, Nuclear Research Center, Dokki, Giza, Egypt.

A commercial potting soil was used (density: 450 g/L; moisture: 12–15%; organic matter: 75–80%; pH 5.9–6.3; TDS 350–500 ppm), composed of coco-peat, moss, perlite, humic acid, plant compost, and vermiculite. Pots (30 × 40 cm) were established in three replicates per treatment. In each pot, 35 radish seeds (*Raphanus sativus* cv. Balady) were sown at 5 cm spacing and consistently irrigated with deionized water to maintain constant soil humidity.

Al-MOF and Ag_2_CrO_4_/Al-MOF were dispersed in distilled water (pH 7.0, without surfactants) and applied as a foliar spray at a concentration of 10 mg/L. Treatments began 28 days after sowing and were performed twice with a 10-day interval. Applications were conducted in the early morning (before 9:00 a.m.) using a pressurized hand sprayer to ensure small droplet sizes and minimize wind interference. To ensure adequate coverage, each row of plants was sprayed from both sides.

The experimental design consisted of six treatment combinations: two gamma irradiation doses (0 and 10 Gy) and three foliar spray variants (Control, Al-MOF, and Ag_2_CrO_4_/Al-MOF). Plants were sampled before spraying (0 DAS) and at 3, 6, and 9 days after spraying (DAS) during the vegetative growth stage. Harvested plants were submerged in water and lightly shaken to remove soil particles before being processed for growth performance, physiological assessments, and molecular analysis. Results are presented as the average of three replicates, with 15 seedlings analyzed per treatment.

### Determination of metal content

The harvested *Raphanus sativus* leaves were washed with distilled water to remove dust and surface particulates. The analyzed tissues were then cut into small pieces and oven-dried at 70 °C until a constant weight was achieved. The dried samples were ground into a fine powder and stored in polyethylene bags until acid digestion.

The wet ashing method was performed according to the protocol described by Chapman and Pratt [25]. Ground plant material was digested using an acid mixture of concentrated nitric acid (HNO_3_), sulfuric acid (H_2_SO_4_), and 60–62% perchloric acid (HClO_4_) in a 10:1:4 ratio, respectively. Digestion continued until the solution became clear, indicating complete oxidation of organic matter. The resulting samples were then diluted with deionized distilled water, filtered through acid-washed filter paper, and adjusted to a known volume. The filtrates were stored in stoppered bottles for elemental analysis.

Aluminum (Al) and Chromium (Cr) concentrations (expressed as mg/kg dry weight) were determined using an iCAP™ 7400 ICP-OES Analyzer. All ICP-OES measurements were performed in triplicate to ensure analytical precision.

### Growth parameters

Growth performance was evaluated by determining root length (cm), shoot height (cm), the root-to-shoot length ratio, leaf area (cm^2^), and overall plant phenotype. Samples were harvested from the inner rows of each treatment, with five plants randomly selected per replicate.

Measurements for root length, shoot height, leaf number per plant, and leaf area were conducted concurrently. Root length was determined by averaging the length of the longest roots. Shoot height was measured as the distance from the base of the main stem to the tip of the third or fourth emerging leaf. Leaf area was calculated according to the formula established by Wang et al. [26].

### Chlorophyll contents

Chlorophyll content was measured using the second fully expanded, unshaded leaf from the apex. A portable SPAD-502 Chlorophyll Meter (Minolta Co., Ltd.) was utilized to determine the Chlorophyll Content Index (CCI) through a non-destructive method, following the protocol described by Vishwakarma et al. [27].

### Ascorbic acid determination

Ascorbic acid was estimated according to the method described by Bajaj and Kaur [28]. Reagents: 1- (5% w/ v) ammonium molybdate solution. 2- (0.05 M) freshly prepared oxalic acid solution containing 0. 2 mM EDTA. 3- (5% v/v) sulphuric acid. 4- Metaphosphoric acid -acetic acid solution. Fifteen gm of metaphosphoric acid pellets in (40 ml) acetic acid and (200 ml) distilled water, then diluted (to 500 ml) with distilled water then filtered. In the refrigerator, that reagent can be held over for 3 days. 5- In the oxalic acid-EDTA solution, the standard L-ascorbic acid solution (0.1% w/v) was freshly prepared. Five gm of fresh samples was used for extraction in a 100 ml of oxalic acid -EDTA solution. Then the extract was filtered across filter-papers followed by centrifugation. The (5 ml) aliquot was transmitted into a (25 ml) calibration flask. After 15 min the absorbance of the blue solution was measured at 760 nm. The ascorbic acid concentration (expressed as µg g^− 1^ F. wt.) was calculated using the standard curve.

### Total phenol determination

The content of total phenol was assayed through the procedure suggested by Waterhouse [29] using the Folin - Ciocalteu reagent. An (0. 5 ml) aliquot of the extract was added to tubes containing (2. 5 ml) 10% Folin - Ciocalteu solution (v /v) followed by the addition of (2 mL) 4% sodium carbonate (v/v), vortexed and rest for 120 min protected from light. Spectrophotometrically at 685 nm, the blue color developed by the reduction of the Folin - Ciocalteu reagent by phenols was measured. The calculation of the total phenol content was derived from the gallic acid standard curve line. The results were expressed in µg of gallic acid equivalent per gram of sample (µg GAE.g ^-1^ F. wt.). Absorbance measurements for the determination of total phenol and ascorbic acid content were performed using a Tomos UV-1800 UV-VIS Spectrophotometer. A glass cell with a 1.0 cm optical path length was utilized for all measurements.

### DNA isolation

Genomic DNA was extracted from the freshly shoot area (leaf and stem) of *Raphanus sativus* on the ninth DAS by DNeasy plant mini kit (bio basic). The quality of DNA was assessed using absorbance ratios A260/A280 via a UV spectrophotometer, indicating purity when the ratio falls between 1.8 and 2.0. Furthermore, a qualitative assessment of DNA samples was conducted utilizing electrophoresis on 1% agarose gel containing ethidium bromide.

### Primers design

For the molecular evaluation of *Raphanus sativus* treatments, genomic DNA was utilized as a template for Polymerase Chain Reaction (PCR) amplification with 10 SCoT primers. In contrast, SCoT primers were sourced from Biobasic Com. and were built using consensus sequences from prior research [30–32]. Dataset I, based on genes with high expression levels as stated by Sawant et al. [33]. was used to generate all 18-mer SCoT primers (Table 1).

**Table 1:** List of ten SCoT primer sequences utilized in *Raphanus sativus* under different treatments on 9DAS

Primer	Sequences
S CoT-1	5’-ACGAC*ATG*GCG ACCACGC-3’
S CoT-2	5’-ACC*ATG*GCTA CCACCGGC-3’
S CoT-3	5’-ACGAC*ATG*G CGACCCACA-3’
S CoT-4	5’-AC C*ATG*G CTACCACCGCA-3’
S CoT-5	5’-CA *ATG*GCTACCACTAGCG-3’
S CoT-7	5’-ACA*ATG*GCTACCAC TGAC-3’
S CoT-9	5’-ACA*ATG*GCTACC ACTGCC-3’
S CoT-10	5’-ACA*ATG*GC TACCACCAGC-3’
S CoT-12	5’-CAACA*ATG*GCT ACCACCG-3’
S CoT-14	5’-ACC*ATG*GCTACCA GCGCG-3’

### Polymerase chain reaction

Amplification reactions for SCoT techniques were conducted according to the methodologies outlined by Fathi et al. [34]. and Xiong et al. [24]., utilizing the Techni TC-512 Thermal Cycler as follows: One cycle at 94 °C for 4 min followed by 40 cycles of 1 min at 94 °C, 1 min at annealing temperature 57 °C for 2 min at 72 °C, followed by 72 °C for 10 min, the reaction was finally stored at 4 °C.

### Gel electrophoresis

Amplified products were loaded and separated on a 1.5% agarose gel containing ethidium bromide, with 100 bp to 1 kb ladder markers. The run was conducted for approximately 30 min at 100 V in a BioRad mini submarine gel.

### Gel reading and analysis

Photos of DNA banding patterns were captured using the Bio-1D Gel Documentation system and evaluated with GelAnalyzer3 software, which scored distinct amplicons as present (1) or absent (0) for each primer, resulting in a binary data matrix. DNA profiles were generated from this matrix using SCoT procedure as per Adhikari et al. [35]. Molecular distances DM (Dissimilarity) were calculated using the Dice coefficient [36] based on binary data matrices, and agglomerative hierarchical clustering (AHC) analysis was conducted via the Unweighted Pair-Group average UPGMA method using XLSTAT.7 software.

### Statistical analysis

The experiment was conducted using a completely randomized design (CRD) with six treatments and three replications. Results are expressed as the mean ± standard error (SE). To determine the degree of significance between treatments, data were subjected to a two-way analysis of variance (ANOVA) using IBM SPSS Statistics version 26.0 (IBM Corp., Armonk, NY). Significant differences among treatment means were identified at *p* ≤ 0.05 and the differences between means were determined using Duncan’s test. Additionally, an independent T-test was employed to compare the means within the same treatment across different time points (0 DAS and 9 DAS), with significance also established at *p* ≤ 0.05.

## Results

### Characterization of fertilizer material

**Fig. 1:**
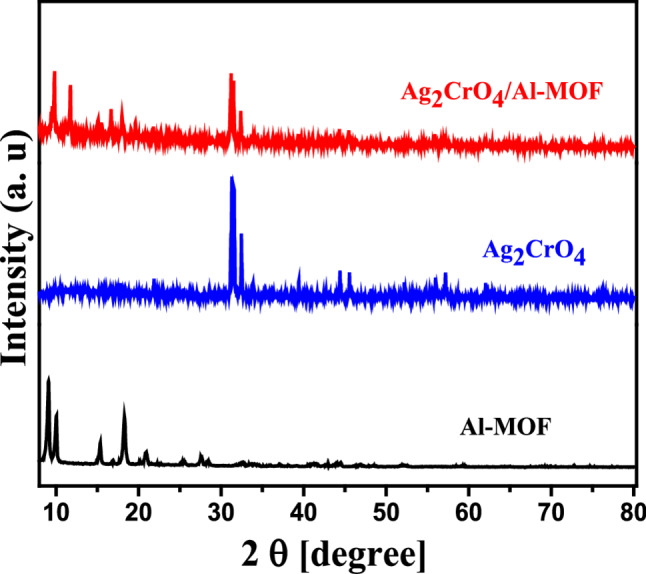
XRD patterns of Ag_2_CrO_4_, Al-MOF and the Ag_2_CrO_4_/Al-MOF composite

The crystal structures of Ag_2_CrO_4_, Al-MOF, and the Ag_2_CrO_4_/Al-MOF composite were confirmed via XRD analysis. As shown in Fig. 1, characteristic diffraction peaks for the Al-MOF sample were observed at 2 theta = 9.7°, 11.6°, 15.4°, 16.7°, and 19.5°. Furthermore, all pronounced characteristic peaks of Ag_2_CrO_4_ were well-indexed and consistent with the orthorhombic phase of Ag_2_CrO_4_. Specifically, diffraction peaks at 2 theta = 31.1°, 31.4°, 32.3°, 45.4°, and 55.8° are attributed to the (031), (211), (002), (240), and (242) planes, respectively, which correspond to the orthorhombic phase of Ag_2_CrO_4_ (JCPDS No. 26–0952). In the Ag_2_CrO_4_/Al-MOF composite spectrum, several peaks were observed at 31.1°, 31.5°, 32.5°, 39.5°, 44.4°, and 45.2° due to the integration of Ag_2_CrO_4_, appearing alongside the significant peaks characteristic of the Al-MOF framework.

**Fig. 2:**
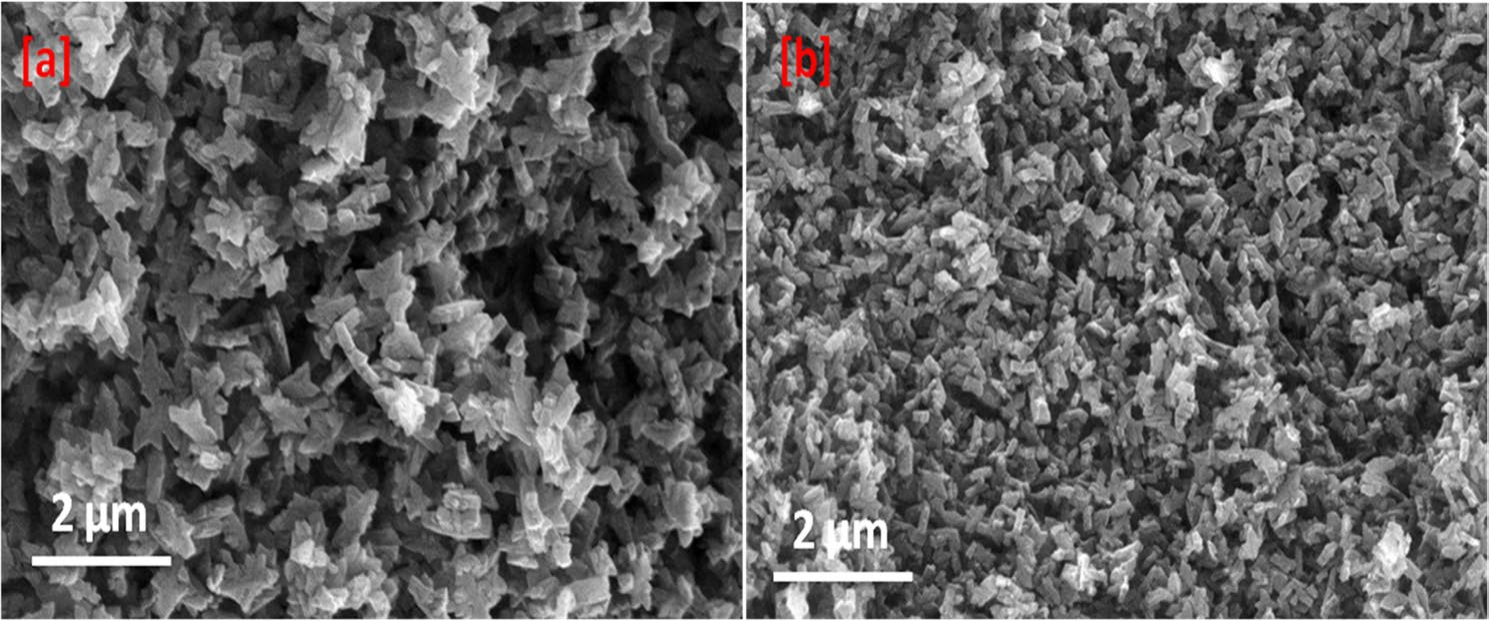
SEM images of (**a**) Al-MOF, and (**b**) Ag_2_CrO_4_/Al-MOF

To characterize the morphology of Ag_2_CrO_4_, Al-MOF, and the Ag_2_CrO_4_/Al-MOF composite, microscopic analysis was performed using Scanning Electron Microscopy (SEM); representative images of these materials are presented in Fig. 2. The Al-MOF substrate displays characteristic rod-like nanocrystals (Fig. 2a). In the composite Ag_2_CrO_4_/Al-MOF (Fig. 2b), the observed structure retains a morphology similar to that of the Al-MOF, suggesting that the Ag_2_CrO_4_ nanoparticles successfully grew on or integrated into the surface of the Al-MOF framework. Ag_2_CrO_4_ nanoparticles showed orthorhombic crystal structure with an average crystallite size of approximately 20–38 nm. Ag_2_CrO_4_ has a negative surface charge, ranging from − 17 mV to -20 mV. This negative charge contributes to its interaction with positively charged MOF surfaces. The size of Al-MOF crystals was 100 nm, with zeta potential between − 10 mV and + 10 mV. Ag_2_CrO_4_/Al-MOF composite size showed a slight increase in the overall hydrodynamic diameter (150 nm). The incorporation of Ag_2_CrO_4_ on to MOF shifts the zeta potential to more negative values (-25 mV), which is beneficial for dispersing the composite in water for plant applications.

**Fig. 3:**
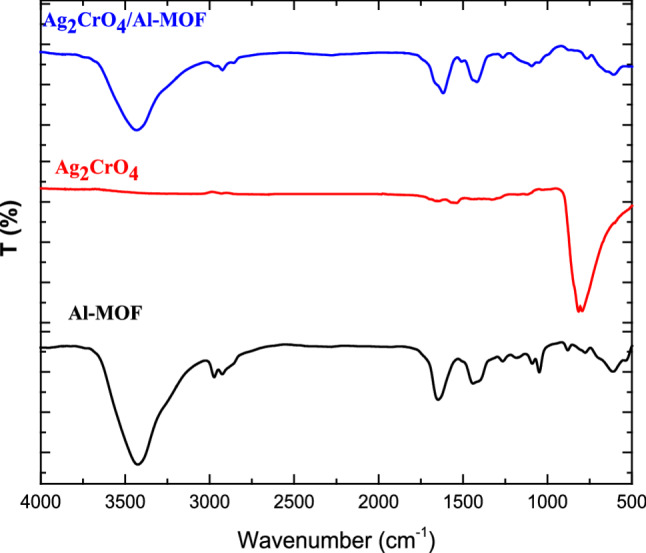
FTIR of Ag_2_CrO_4_, Al-MOF and the Ag_2_CrO_4_/Al-MOF

The FTIR spectra of Ag_2_CrO_4_, Al-MOF, and the Ag_2_CrO_4_/Al-MOF composite are shown in Fig. 3. For the Al-MOF, characteristic signals are observed at 3070 cm^-1^, corresponding to the C-H stretching of the aromatic rings, and at 1660 cm^-1^, attributed to the C = O stretching vibrations. The peaks at 1370 cm^-1^ correspond to the asymmetric and symmetric stretching of the carboxylate groups. Additionally, the peaks identified at 1148, 1105, and 1016 cm^-1^ are attributed to C-C vibrations. In the spectrum of pure Ag_2_CrO_4_, the strong absorption peak at 803 cm^-1^ is related to the Cr-O stretching vibrations of the chromate groups. For the Ag_2_CrO_4_/Al-MOF composite, all primary peaks characteristic of Al-MOF are preserved, with the appearance of an additional peak at 818 cm^-1^. This peak originates from the Cr-O stretching vibrations, confirming the successful integration of Ag_2_CrO_4_ into the MOF framework.

### Metal content

**Fig. 4:**
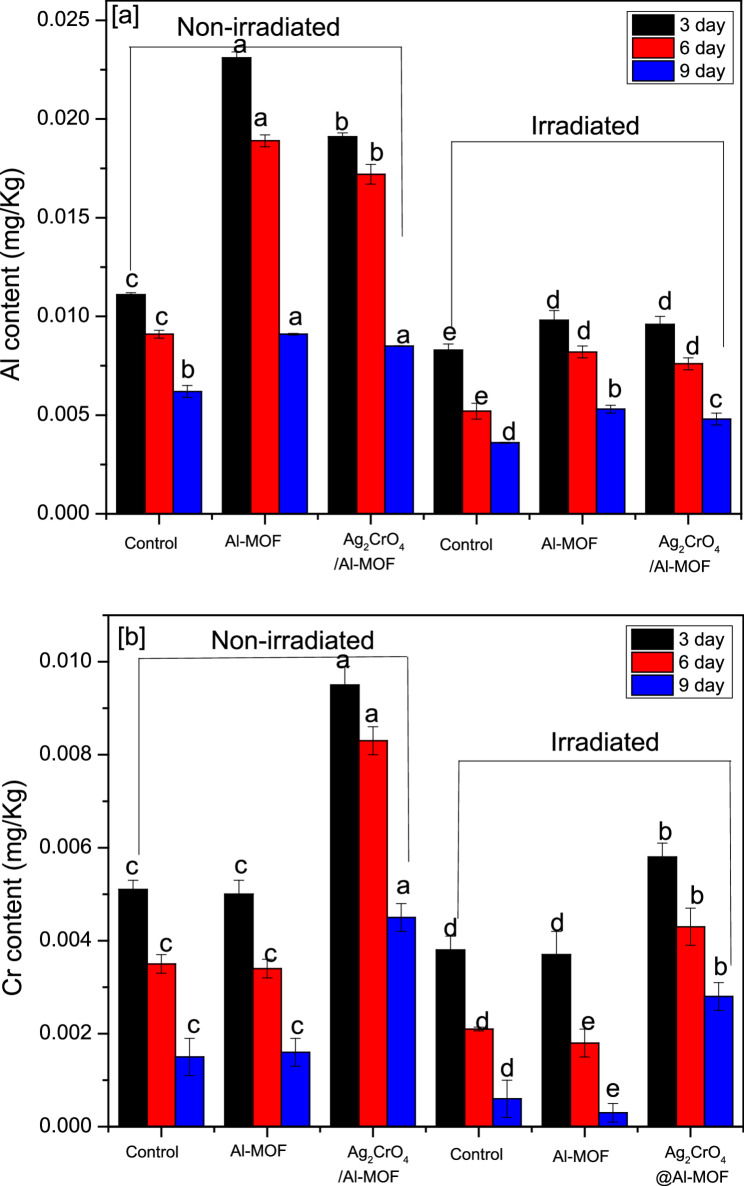
Temporal changes in metal accumulation in *Raphanus sativus* leaves: (**a**) Aluminum (Al^3+^) content and (**b**) Chromium (Cr^3+^) content in mg/kg. Data represents various treatment groups evaluated at 3, 6, and 9 DAS (Days After Spray)

The concentrations of Al^3+^ and Cr^3+^ in *Raphanus sativus* leaves under all experimental treatments, including the control, were negligible, as illustrated in Fig. 4; Table 2. Notably, the Cr^3+^ content was significantly lower than the permissible limit of 1.3 mg/kg established by the WHO (1996) for plants. Furthermore, the recorded levels remained well below the established oral reference dose for Al^3+^, which is 1.0 mg/kg/day [37].

**Table 2:** Aluminum (Al^3+^) and Chromium (Cr^3+^) concentrations in *Raphanus sativus* leaves at 3, 6, and 9 days after spray (DAS) under various treatments

	Leaves content of Al^3+^ and Cr^3+^ mg/KG						
		3DAS		6DAS		9DAS	
Treatment		Al^3+^	Cr^3+^	Al^3+^	Cr^3+^	Al^3+^	Cr^3+^
Non-irradiated	control	0.0111 c	0.0051 c	0.0091 c	0.0035 c	0.0062 b	0.0015 c
	Al-MOF	0.0231 a	0.0050 c	0.0189 a	0.0034 c	0.0091 a	0.0016 c
	Ag_2_CrO_4_/Al-MOF	0.0191 b	0.0095 a	0.0172 b	0.0083 a	0.0085 a	0.0045 a
Irradiated	control	0.0083 e	0.0038 d	0.0052 e	0.0021 d	0.0036 d	0.0006 d
	Al-MOF	0.0098 d	0.0037 d	0.0082 d	0.0018 e	0.0053 b	0.0003 e
	Ag_2_CrO_4_/Al-MOF	0.0096 d	0.0058 b	0.0076 d	0.0043 b	0.0048 c	0.0028 b

Mean (*n* = 3) different letters in the same column are statistically different (*p* ≤ 0.05) and represent the differences between treatments. *DAS* Day after spray

### Growth parameters

#### Plant phenotype

**Fig. 5:**
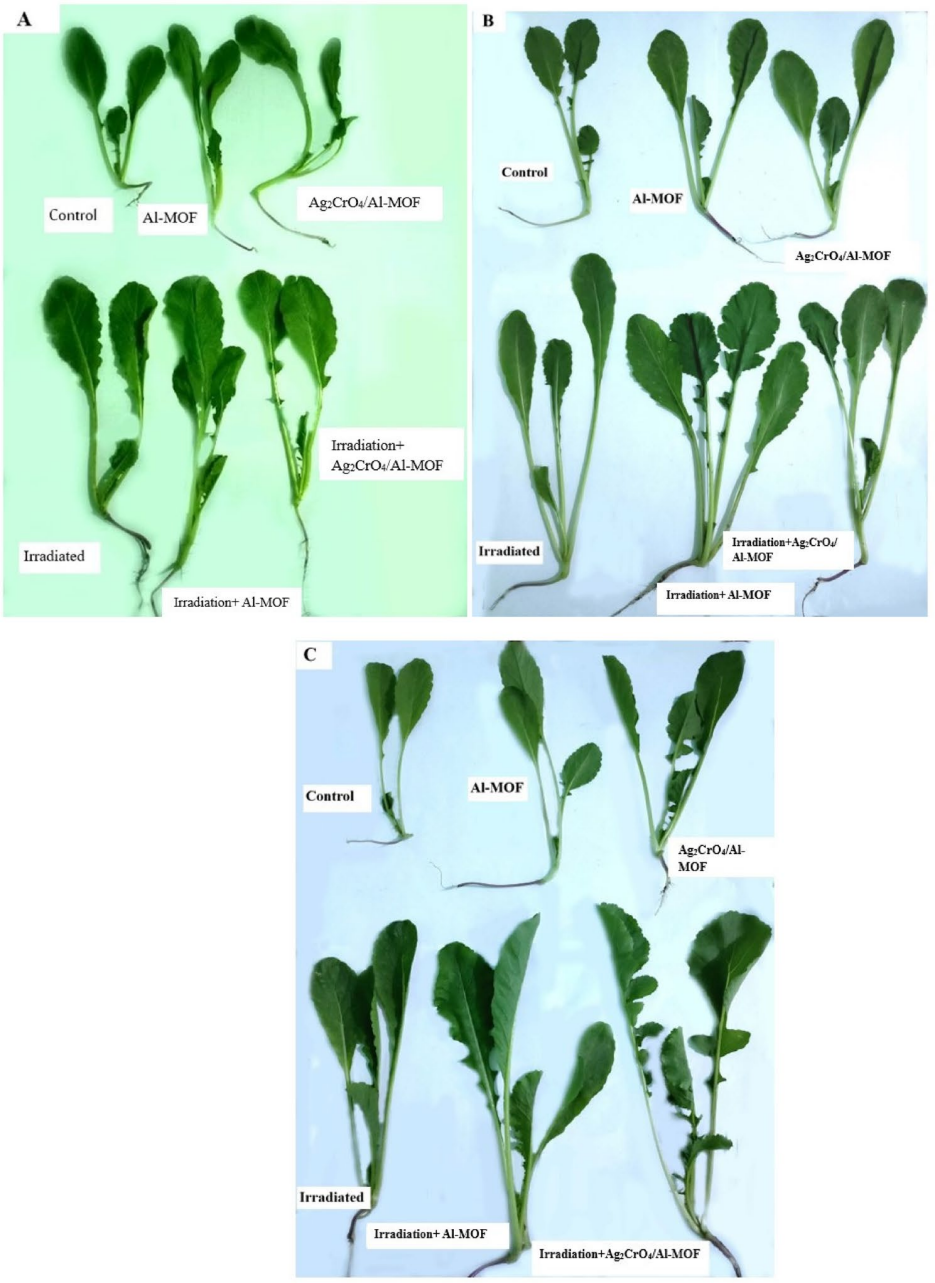
Morphological response and phenotype of *Raphanus sativus* across treatment groups, recorded at three intervals: 3 DAS (**A**), 6 DAS (**B**), and 9 DAS **C**

Plant phenotype refers to the observable characteristics of a plant, resulting from the interaction of its genetic makeup (genotype) and the environment it grows in. Plant phenotype is a description of a plant’s observable traits (physical form). *Raphanus sativus* phenotypes are significantly affected by various treatments as shown in Fig. 5.

#### Number of leaves/ plant

All *Raphanus sativus* plants at the starting point 0DAS presented normal three leaves phenotype except with irradiation+Ag_2_CrO_4_/Al-MOF treatment that exhibited four leaves phenotype. After 6DAS, four leaves appeared in all treatments that included irradiation. However, it was worth mentioning that after 9DAS all *Raphanus sativus* plants germinated from irradiated seeds as well as Ag_2_CrO_4_/Al-MOF treatment showed improved phenotypic appearances (four leaves) relative to non-irradiated ones (Table S1).

#### Shoot height

Both gamma irradiation and the synthesized MOFs exerted significant, time-dependent influences on the shoot height of *Raphanus sativus*. As detailed in Table 3, all experimental groups—including irradiation alone Al-MOF and Ag_2_CrO_4_/Al-MOF—demonstrated a clear growth-promoting effect compared to the control. Notably, the magnitude of these developmental gains varied significantly across the study period, with the Ag_2_CrO_4_/Al-MOF treatment, particularly when combined with irradiation, yielding the most pronounced increases in vertical growth.

**Table 3:** Effects of various treatments on the shoot height (cm) of *Raphanus sativus* at 0, 3, 6, and 9 days after spray (DAS)

			Shoot height (cm)		
Treatment		0DAS	3DAS	6DAS	9DAS
Non-irradiated	Control	13.5 ± 0.41 d	16.0 ± 0.34 c	17.3 ± 0.83 d	19.0 ± 0.12 c*
	Al-MOF	16.0 ± 0.21 c	18.0 ± 0.79 bc	19.5 ± 0.77 cd	21.0 ± 0.38 b*
	Ag_2_CrO_4_/Al-MOF	15.5 ± 0.49 c	18.0 ± 0.33 bc	20.2 ± 0.39 bc	21.0 ± 0.61 b*
Irradiated	Control	19.5 ± 0.47 b	20.0 ± 0.33 b	22.5 ± 0.74 ab	25.5 ± 0.14 a*
	Al-MOF	22.5 ± 0.41 a	22.5 ± 0.82 a	24.5 ± 0.41 a	27.0 ± 0.56 a*
	Ag_2_CrO_4_/Al-MOF	19.5 ± 0.76 b	23.0 ± 0.21 a	25.0 ± 0.94 a	26.0 ± 0.85 a*

Mean (*n* = 3) ± standard error. Different letters in the same column are statistically different (*p* ≤ 0.05) and represent the differences between treatments across the same time point. *Represent the significant differences (*p* ≤ 0.05) between means (in the same row) within the same treatment across different time points 0DAS and 9DAS. *DAS* Day after spray

#### Root length

MOF-nanoparticles with or without irradiation showed significant influences on root length in plants, The effects were clearly visible during the period from 3DAS to 6DAS. All treatments on the 9DAS have been significantly promoted root growth over control except with Al-MOF as shown in Table 4.

**Table 4:** Impact of various treatments on the root length (cm) of *Raphanus sativus* at 0, 3, 6, and 9 days after spray (DAS)

			Root length (cm)		
Treatment		0DAS	3DAS	6DAS	9DAS
Non-irradiated	Control	7.0 ± 0.29 c	7.2 ± 0.36 e	8.0 ± 0.51 d	10.5 ± 0.39 b*
	Al-MOF	8.5 ± 0.41 b	9.6 ± 0.08 bc	10.3 ± 0.58 bc	11.7 ± 0.42 ab*
	Ag_2_CrO_4_/Al-MOF	6.5 ± 0.30 c	9.0 ± 0.25 cd	10.0 ± 0.68 bc	12.6 ± 0.21 a*
Irradiated	Control	7.0 ± 0.22 c	8.0 ± 0.41 de	9.5 ± 0.37 cd	13.0 ± 0.21 a*
	Al-MOF	9.4 ± 0.45 ab	10.7 ± 0.61 b	11.5 ± 0.28 ab	12.3 ± 0.34 a*
	Ag_2_CrO_4_/Al-MOF	10.0 ± 0.19 a	12.3 ± 0.05 a	12.5 ± 0.17 a	13.0 ± 0.62 a*

Mean (*n* = 3) ± standard error. Different letters in the same column are statistically different (*p* ≤ 0.05) and represent the differences between treatments across the same time point. *Represent the significant differences (*p* ≤ 0.05) between means (in the same row) within the same treatment across different time points 0DAS and 9DAS. *DAS* Day after spray

#### Root to shoot length ratio

The root-to-shoot length ratio in plants is a measure of how much biomass is allocated to the roots compared to the above-ground portion (shoot). MOF-nanoparticles with or without irradiation treatments showed influence on this ratio. The effects on root to shoot ratio (Table 5) are complex and depend on various factors, including MOF-nanoparticle type, irradiation and MOF single or double effects, and duration after spraying.

**Table 5:** Root-to-shoot length ratio of *Raphanus sativus* under various treatments at 0, 3, 6, and 9 days after spray (DAS)

		Root to shoot length ratio			
Treatment		0DAS	3DAS	6DAS	9DAS
Non-irradiated	Control	0.52 ± 0.04 a	0.45 ± 0.01 ab	0.46 ± 0.01 a	0.55 ± 0.02 a
	Al-MOF	0.53 ± 0.03 a	0.54 ± 0.02 a	0.53 ± 0.05 a	0.56 ± 0.01 a
	Ag_2_CrO_4_/Al-MOF	0.42 ± 0.01 b	0.50 ± 0.02 b	0.50 ± 0.04 a	0.60 ± 0.01 a*
Irradiated	Control	0.36 ± 0.02 b	0.40 ± 0.03 b	0.42 ± 0.02 a	0.51 ± 0.01 abc*
	Al-MOF	0.42 ± 0.01 b	0.48 ± 0.04 ab	0.47 ± 0.02 a	0.46 ± 0.02 c
	Ag_2_CrO_4_/Al-MOF	0.52 ± 0.03 a	0.53 ± 0.01 a	0.50 ± 0.01 a	0.50 ± 0.04 bc

Mean (*n* = 3) ± standard error. Different letters in the same column are statistically different (*p* ≤ 0.05) and represent the differences between treatments across the same time point. *Represent the significant differences (*p* ≤ 0.05) between means (in the same row) within the same treatment across different time points 0DAS and 9DAS. *DAS* Day after spray

#### Leaf area

The leaf area was improved during the experiment depending on various factors including time, irradiation as well as the type of MOF-nanoparticles. The leaf area at the end of the experiment increased 80%, 133% and 132% by treatment with irradiation, irradiation + Al-MOF and irradiation+Ag_2_CrO_4_/Al-MOF, respectively. Enhanced leaf area might be potentially through improved nutrient uptake or stress mitigation as shown in Table 6, Figure S1.

**Table 6:** Effects of different treatments on the leaf area (cm^2^) of *Raphanus sativus* at 0, 3, 6, and 9 days after spray (DAS)

			Leaf area (cm^2^)		
Treatment		0DAS	3DAS	6DAS	9DAS
Non-irradiated	Control	16.07 ± 0.25 e	17.95 ± 0.19 f	21.30 ± 0.79 d	23.70 ± 0.16 e*
	Al-MOF	22.37 ± 0.51 d	26.69 ± 0.18 e	30.93 ± 0.73 c	37.02 ± 0.35 c*
	Ag_2_CrO_4_/Al-MOF	25.23 ± 0.71 c	30.07 ± 0.75 d	32.42 ± 0.40 c	34.66 ± 0.59 d*
Irradiated	Control	36.07 ± 0.70 b	36.33 ± 0.21 c	40.44 ± 0.62 b	42.66 ± 0.23 b*
	Al-MOF	45.26 ± 0.23 a	48.47 ± 0.74 b	51.37 ± 0.57 a	55.22 ± 0.51 a*
	Ag_2_CrO_4_/Al-MOF	46.51 ± 0.57 a	50.56 ± 0.32 a	52.76 ± 0.93 a	55.08 ± 0.41 a*

Mean (*n* = 3) ± standard error. Different letters in the same column are statistically different (*p* ≤ 0.05) and represent the differences between treatments across the same time point. *Represent the significant differences (*p* ≤ 0.05) between means (in the same row) within the same treatment across different time points 0DAS and 9DAS. *DAS* Day after spray

### Chlorophyll content index

The efficacy of irradiation and/or MOFs on plants were evaluated on the basis of the index of greenness of the *Raphanus sativus* leaves. Table 7 presents the CCI of *Raphanus sativus* leaves. All *Raphanus sativus* plants at the starting point 0DAS presented non-significant differences between treatments with the control. After 3DAS, some few significant differences appeared. Ag_2_CrO_4_/Al-MOF sprayed *Raphanus sativus* showed the highest CCI that differed significantly form the control as well as from the Al-MOF-sprayed plants. After 6DAS, the differences were appeared in a way that is readily distinguishable clearly between different treatments. The highest chlorophyll content was detected in Ag_2_CrO_4_/Al-MOF sprayed plants regardless of they were irradiated or not as well as the chlorophyll of irradiated *Raphanus sativus*. After 9DAS, the chlorophyll content became convergent between treatments with the lowest content in control that did not differ significantly with Al-MOF-sprayed plants even they were irradiated or not.

**Table 7:** Chlorophyll Content Index (CCI) in *Raphanus sativus* under various treatments at 0, 3, 6, and 9 days after spray (DAS)

			Chlorophyll (CCI)		
Treatment		0DAS	3DAS	6DAS	9DAS
Non-irradiated	Control	9.5 ± 0.17 a	9.6 ± 0.22 b	9.0 ± 0.22 c	9.0 ± 0.22 b
	Al-MOF	9.5 ± 0.17 a	9.6 ± 0.35 b	9.6 ± 0.27 c	10.0 ± 0.22 ab
	Ag_2_CrO_4_/Al-MOF	10.3 ± 0.15 a	10.4 ± 0.42 ab	10.8 ± 0.07 a	10.8 ± 0.50 a
Irradiated	Control	10.5 ± 0.67 a	10.7 ± 0.25 ab	10.6 ± 0.12 ab	10.7 ± 0.14 a
	Al-MOF	9.7 ± 0.36 a	9.9 ± 0.28 b	9.8 ± 0.40 bc	10.1 ± 0.53 ab
	Ag_2_CrO_4_/Al- MOF	11.1 ± 0.47 a	11.3 ± 0.26 a	11.2 ± 0.28 a	11.1 ± 0.17 a

Mean (*n* = 3) ± standard error. Different letters in the same column are statistically different (*p* ≤ 0.05) and represent the differences between treatments across the same time point. *Represent the significant differences (*p* ≤ 0.05) between means (in the same row) within the same treatment across different time points 0DAS and 9DAS. *DAS* Day after spray

### Ascorbic acid

**Fig. 6:**
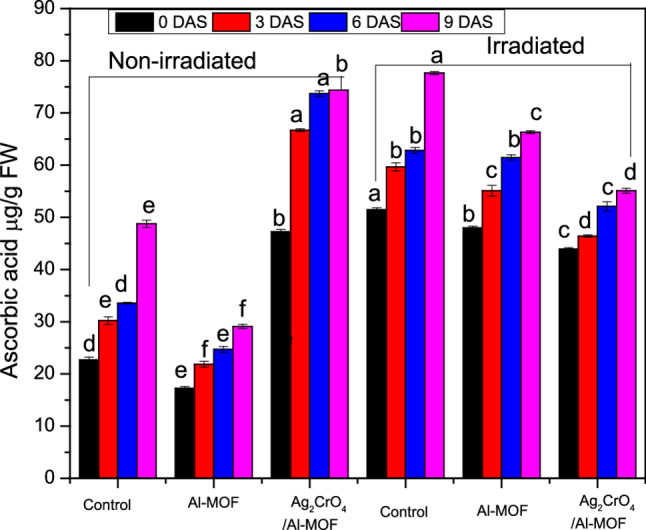
Ascorbic acid concentration (µg/g fresh weight) in *Raphanus sativus* under various treatments at 0, 3, 6, and 9 days after spray (DAS)

Table 8; Fig. 6 present *Raphanus sativus* leaves content of ascorbic acid. The differences in the ascorbic acid content were appeared clearly between different treatments from the 0DAS, except between Al-MOF and irradiation + Al-MOF treatments. The highest ascorbic acid content was detected as a result of irradiation while the lowest content was due to Al-MOF treatment. After 3 days, the ascorbic acid content differed significantly between all treatments until the 9DAS. Till the 9DAS, the highest ascorbic acid content was detected with irradiation treatments followed by Ag_2_CrO_4_/Al-MOF then irradiation + Al-MOF treatments. On the other side, the ascorbic acid content through days showed significant differences with the 0DAS content due to all treatments (Figure S3).

**Table 8:** Ascorbic acid content (µg/g fresh weight) in *Raphanus sativus* under different treatments at 0, 3, 6, and 9 days after spray (DAS)

		Ascorbic acid µg /g fresh wt.			
Treatment		0DAS	3DAS	6DAS	9DAS
Non-irradiated	control	22.72 ± 0.48 d	30.21 ± 0.76 e	33.57 ± 0.15 d	48.76 ± 0.69 e*
	Al-MOF	17.26 ± 0.33 e	21.85 ± 0.57 f	24.70 ± 0.55 e	29.12 ± 0.40 f*
	Ag_2_CrO_4_/Al-MOF	47.28 ± 0.41 b	66.70 ± 0.29 a	73.71 ± 0.53 a	74.37 ± 0.03 b*
Irradiated	control	51.44 ± 0.38 a	59.67 ± 0.76 b	62.80 ± 0.57 b	77.63 ± 0.27 a*
	Al-MOF	47.95 ± 0.36 b	55.12 ± 1.02 c	61.48 ± 0.50 b	66.31 ± 0.29 c*
	Ag_2_CrO_4_/Al-MOF	43.95 ± 0.22 c	46.41 ± 0.23 d	52.11 ± 0.87 c	55.08 ± 0.47 d*

Mean (*n* = 3) ± standard error. Different letters in the same column are statistically different (*p* ≤ 0.05) and represent the differences between treatments across the same time point. *Represent the significant differences (*p* ≤ 0.05) between means (in the same row) within the same treatment across different time points 0DAS and 9DAS. *DAS* Day after spray

### Total phenol

**Fig. 7:**
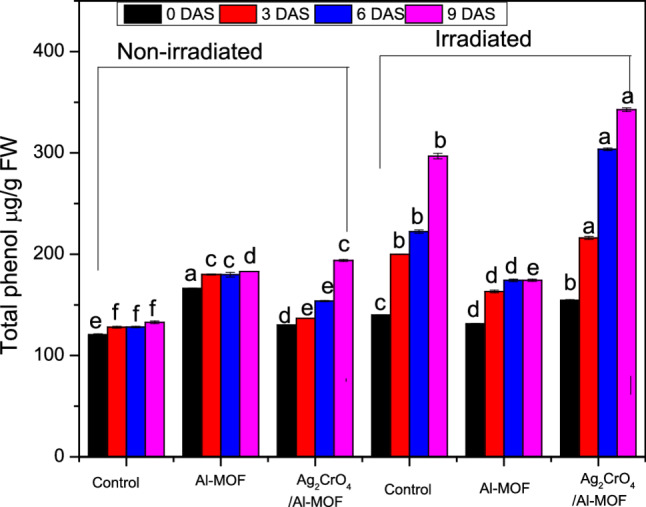
Total phenolic content (µg GAE /g fresh weight) in *Raphanus sativus* under various treatments at 0, 3, 6, and 9 days after spray (DAS)

Total phenol in *Raphanus sativus* can be significantly affected by the application of irradiation and/ or MOF-nanoparticles, with both increases and decreases observed depending on the type of treatments used as shown in Fig. 7; Table 9. Control plants recorded the least phenol content through the experiment. Al-MOF sprayed plants showed the highest content of phenol followed by irradiation treatments even it treated with Al-MOF, Ag_2_CrO_4_/Al-MOF or not treated, on the 0DAS. Over 3DAS or 6DAS, irradiation combined with Ag_2_CrO_4_/Al-MOF treatments showed the highest content of phenols followed by irradiated non-sprayed plants. After that, came the content of Al-MOF treatments with or even without irradiation. On 9 DAS irradiation+Ag_2_CrO_4_/Al-MOF treatments exhibited the greatest content of phenol followed with unaccompanied irradiation treatment. This was followed by Ag_2_CrO_4_/Al-MOF, Al-MOF and irradiation + Al-MOF, respectively. On the other side, the phenol content increased over days where all treatments showed significant variances when compared with the 0DAS content of phenols (Figure S2).

**Table 9:** Total phenolic content (µg GAE /g fresh weight) in *Raphanus sativus* under various treatments at 0, 3, 6, and 9 days after spray (DAS)

		Total phenol µg GAE /g fresh wt.			
Treatment		0DAS	3DAS	6DAS	9DAS
Non-irradiated	control	120.59 ± 0.91 e	128.01 ± 0.85 f	128.09 ± 0.75 f	132.82 ± 0.31 f*
	Al-MOF	166.27 ± 0.31 a	180.03 ± 0.62 c	179.80 ± 2.26 c	182.95 ± 0.06 d*
	Ag_2_CrO_4_/Al-MOF	130.13 ± 0.05 d	136.76 ± 0.01 e	153.78 ± 0.59 e	193.85 ± 0.93 c*
Irradiated	control	140.03 ± 0.31 c	199.84 ± 0.05 b	222.39 ± 1.57 b	296.80 ± 2.69 b*
	Al-MOF	131.23 ± 0.44 d	163.23 ± 1.34 d	174.15 ± 1.27 d	174.24 ± 1.17 e*
	Ag_2_CrO_4_/Al-MOF	154.72 ± 0.61 b	216.06 ± 1.47 a	303.55 ± 1.24 a	342.60 ± 1.87 a*

Mean (*n* = 3) ± standard error. Different letters in the same column are statistically different (*p* ≤ 0.05) and represent the differences between treatments across the same time point. *Represent the significant differences (*p* ≤ 0.05) between means (in the same row) within the same treatment across different time points 0DAS and 9DAS. *DAS* Day after spray

### Start Codon-Targeted (SCoT) marker analysis

**Fig. 8:**
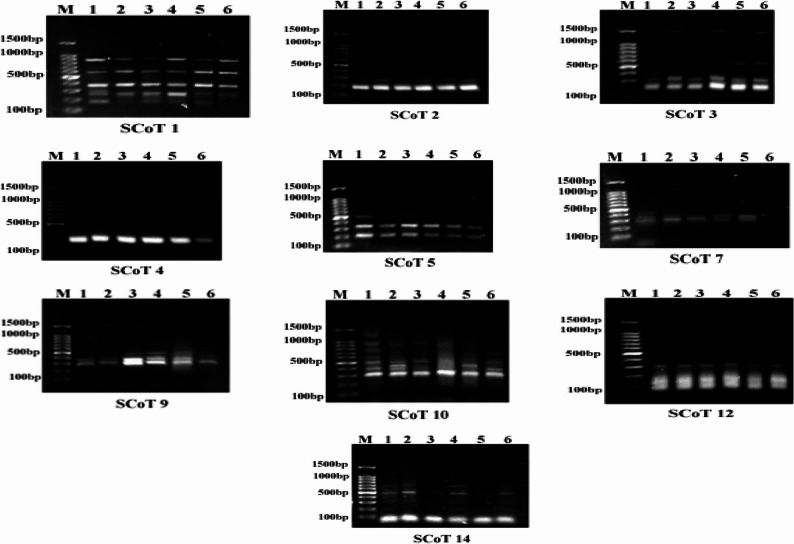
The SCoT amplification profiles patterns obtained using ten primers in the *Raphanus sativus* under different treatments on 9DAS. Lanes: M, DNA ladder; 1, Control; 2, Al-MOF; 3, Ag_2_CrO_4_/Al-MOF; 4, Irradiated; 5, Irradiation + Al-MOF; 6, Irradiation+Ag_2_CrO_4_/Al-MOF

Molecular markers are highly valuable and very useful as shown in Fig. 8; Table 10. SCoT is a polymorphism marker based on short, conserved sequences adjacent to the ATG start codon in plant genes. Ten SCoT primers were employed in evaluating the genetic diversity changes in the *Raphanus sativus* genomes after the exposure of their seeds to the applied dose of gamma irradiation (10 Gy) and spraying plant with MOFs. For SCoT-PCR analysis, a total of 43 DNA bands (24 monomorphic bands, 8 polymorphic bands, and 11 unique bands) were distinguished (Table 10) and Fig. 8. The percentage of polymorphism fluctuated between 33.33% and 66.66% with a mean of 37.7%. The size of the amplified DNA bands varied from 86 to 1875 bp. The primer SCoT-1 revealed the absence of a 275 bp fragment in Al-MOF, Ag_2_CrO_4_/Al-MOF, and irradiated treatments. Fragments of 255 bp were absent in plants treated with irradiation + Al-MOF, and irradiation+Ag_2_CrO_4_/Al-MOF. While the fragment with 185 bp was absent in irradiation+ Ag_2_CrO_4_/Al-MOF in contrast to the control.

**Table 10:** The polymorphism generated by ten SCoT primers used in the *Raphanus sativus* under different treatments on 9 DAS

Primer Name	RBS (bp)	TNB		MB	PB (without unique bands)	UB	PB (with unique bands)	MBF	PIC	Polymorphic %
SCoT 1	185–830	6		3	2	1	3	0.833	0.204	50
SCoT 2	185–385	2		2	-	-	-	1	0	-
SCoT 3	240–1500	4		2	1	1	2	0.791	0.241	50
SCoT 4	300–520	3		2	0	1	1	0.944	0.10	33.33
SCoT 5	200–500	3		3	-	-	-	1	0	-
SCoT 7	275–435	3		1	0	2	2	0.666	0.185	66.66
SCoT 9	300–1865	4		2	1	1	2	0.710	0.181	50
SCoT 10	315–1875	9		3	2	4	6	0.814	0.22	66.66
SCoT 12	86–300	4		4	-	-	-	1	0	-
SCoT 14	320–1365	5		2	2	1	3	0.766	0.233	60
Total		43	24		8	11	19	-	-	-
Mean per primer		4.3	2.4		0.8	1.1	1.9	0.852	0.1366	37.7

(*RBS*) Range of band size (bp), (*TNB*) Total number of Bands, (*MB*) Monomorphic Bands, (*PB without unique bands*) Polymorphic bands without unique bands, (*UB*) Unique bands, (*PB with unique bands*) Polymorphic bands with unique bands, (*MBF*) Mean of band frequency, (*PIC*) Polymorphic information content

In primer SCoT-3, all treated plants detected a fragment at 1500 bp in contrast to the control. Additionally, plants treated with irradiation + Al-MOF and that treated with irradiation+ Ag_2_CrO_4_/Al-MOF showed a fragment at 560 bp in contrast to the control. In the SCoT-4 primer, a fragment at 520 bp was absent for irradiation+Ag_2_CrO_4_/Al-MOF treatments, but it was present in the control. A 435 bp fragment in SCoT-7 was absent for all plant treatments, but it was present in control, while a fragment at 365 bp was absent for irradiation+Ag_2_CrO_4_/Al-MOF treatments, but it was present in the control. Primer SCoT-9 detected one unique fragment at 1865 bp in Al-MOF treatment, while a 520 fragment was present in all treatments except control and Al-MOF treatment.

In the case of SCoT-10, a fragment at 1875 bp was absent for the irradiation + Al-MOF and irradiation+Ag_2_CrO_4_/Al-MOF treatments, while at 945 bp, 500 bp and 400 bp fragments were absent for irradiation+Ag_2_CrO_4_/Al-MOF treatment in contrast to control. On the other hand, control and all treatments except for irradiation + Al-MOF detected a fragment at 730 bp. A 460 bp fragment was identified for Al-MOF and irradiation + Al-MOF, but it was absent in control and other treatments. A1365 bp fragment was found by the SCoT-14 primer in all treatments except for Ag_2_CrO_4_/Al-MOF and irradiation + Al-MOF, while a 765 bp fragment was observed for Al-MOF and irradiated treatments were absent in control and other treatments. Control and all treatments except for Ag_2_CrO_4_/Al-MOF detected a fragment at 320 bp. SCoT-2, SCoT-5, and SCoT-12 have not produced polymorphic fragments. The polymorphic information content (PIC) averaged 0.1366 with values ranging from 0 to 0.241. The SCoT-10 primer produced the highest level of polymorphisms, detecting 6 distinct polymorphic amplification products.

The mean band frequency varied from 0.66 (SCoT-7) to 1.0 (SCoT-2, SCoT-5, and SCoT-12), with an overall average of approximately 0.85. The ten primers generated eleven unique bands—two positive and nine negative markers—which typically arise from structural DNA alterations such as insertions, deletions, transpositions, or strand breaks. These genomic changes can lead to modifications in amino acid sequences and, consequently, alterations in protein structure [38](Table 11). Specifically, six negative molecular markers were identified in the gamma-irradiation + Ag_2_CrO_4_/Al-MOF treatment, with molecular sizes of 185 bp (SCoT-1), 520 bp (SCoT-4), 365 bp (SCoT-7), and 945, 500, and 400 bp (SCoT-10). These findings suggest that the combination of gamma-irradiation and the Ag_2_CrO_4_/Al-MOF composite induced significant genetic variation and DNA instability compared to other treatments. Start Codon Targeted (SCoT) markers are particularly valuable as they are associated with functional genes, allowing for the translation of amplicons into gene-targeted marker systems. As noted by Xiong et al. [24], these are multilocus markers that are highly effective for detecting genetic polymorphism.

**Table 11:** Negative unique markers (NUM) and positive unique markers (PUM) of the ten SCoT primers analyzed in the *Raphanus sativus* under different treatments on 9DAS

Primer	PUM (bp)	NUM (bp)
S CoT- 1	-	185
S CoT-2	-	-
S CoT-3	-	1500
S CoT-4	-	520
S CoT-5	-	-
S CoT-7	435	365
S CoT-9	1865	-
S CoT-10	-	945-730-500-400
S CoT-12	-	-
S CoT-14	-	320
Total	2	9

**Fig. 9:**
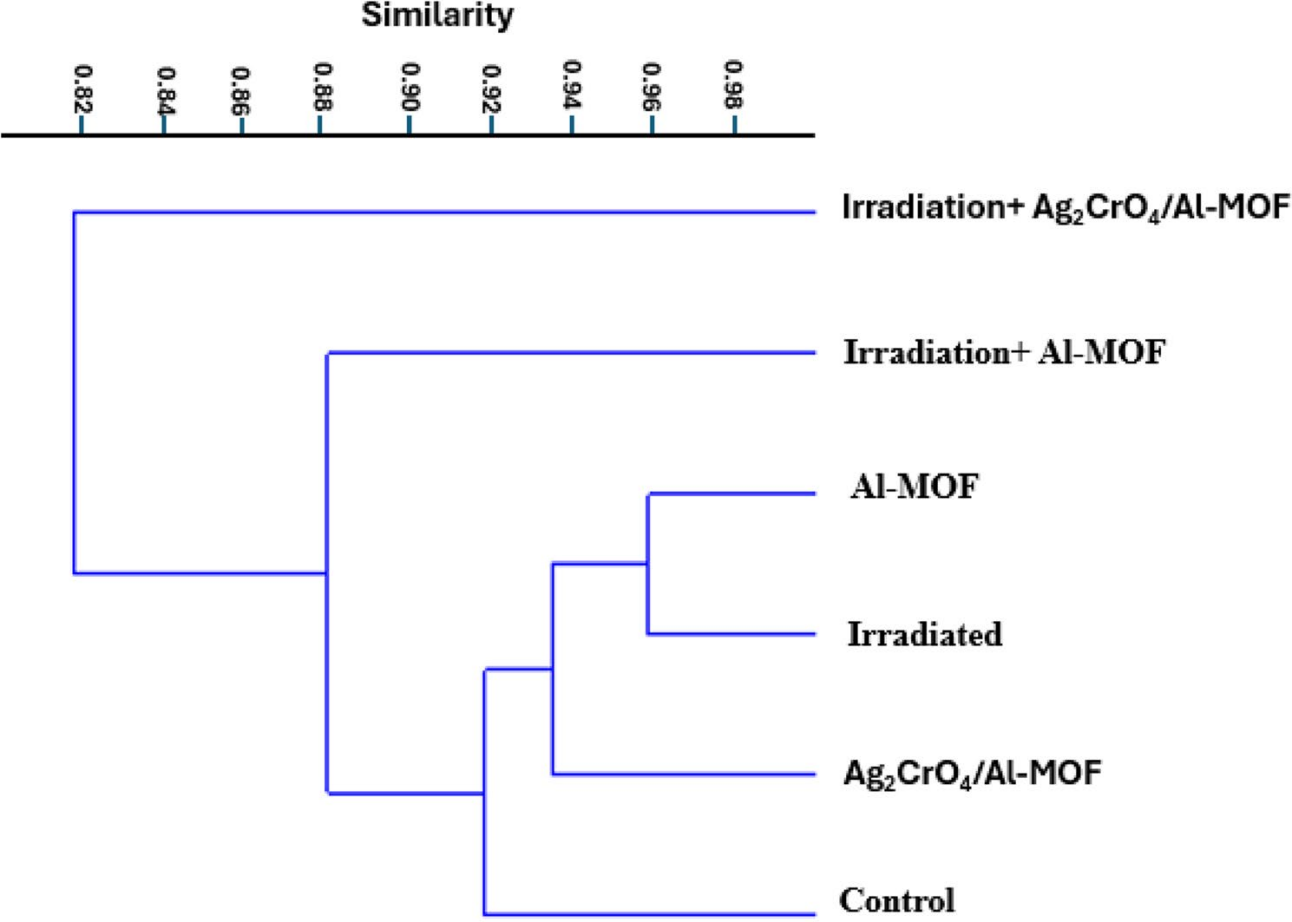
Dendrogram for *Raphanus sativus* under different treatments on 9 DAS created using SCoT data via UPGMA and similarity matrix computed based on Dice’s coefficient

According to Dice’s similarity coefficient (Table 12), the highest genetic similarity was observed between the Al-MOF and gamma-irradiated treatments (0.9589), as well as between the Ag_2_CrO_4_/Al-MOF and gamma-irradiated groups (0.9565). Conversely, the lowest similarity (0.78) was recorded between the Al-MOF treatment and the combined gamma-irradiation + Ag_2_CrO_4_/Al-MOF treatment, further highlighting the unique genetic impact of the combined application. The dendrogram generated from the SCoT data resolved into three primary clusters (Fig. 9). The first cluster exclusively involved the gamma-irradiation + Ag_2_CrO_4_/Al-MOF treatment, while the second cluster comprised the gamma-irradiation + Al-MOF treatment. The third cluster was further subdivided into two distinct subgroups: the first included the independent gamma-irradiated and Al-MOF treatments, while the second group consisted of the Ag_2_CrO_4_/Al-MOF treatment and the control.

**Table 12:** Similarity matrix of *Raphanus sativus* under different treatments on 9DAS utilizing Dice’s coefficient derived from SCoT patterns generated by 10 SCoT primers

Treatment	Control (untreated)	Al-MOF	Ag_2_CrO_4_/Al-MOF	Irradiated	Irradiation + Al-MOF	Irradiation+ Ag_2_CrO_4_/Al-MOF
Control (untreated)	1.0					
Al-MOF	0.91667	1.0				
Ag_2_CrO_4_/Al-MOF	0.91176	0.91429	1.0			
Irradiated	0.92958	0.9589	0.95652	1.0		
Irradiation + Al-MOF	0.86957	0.87324	0.89552	0.88571	1.0	
Irradiation+Ag_2_CrO_4_/Al-MOF	0.8125	0.78788	0.80645	0.83077	0.8571	1.0

## Discussion

Aluminum (Al) is the third most abundant metal in the Earth’s crust. While non-toxic forms of aluminum are prevalent in most soils [39], the solubility and bioavailability of Al^3+^ and Cr^3+^ are negligible within a soil pH range of 5.9–6.3. Under these conditions, Al and Cr primarily exist as insoluble precipitates or are sequestered in stable, non-toxic complexes with organic matter [40]. The biological impact of Al^3+^ and Cr^3^ on plant growth—whether toxic or beneficial—is governed by the principle of hormesis. These effects depend largely on concentration, solubility, and bioavailability (notably, soil pH below 5.0 significantly increases Al solubility), as well as exposure duration, plant species, and developmental stage.

Although Al and Cr can stimulate growth and mitigate both biotic and abiotic stresses, the exact mechanisms by which they mediate these beneficial effects remain largely unknown [40]. While the positive impact of aluminum on plants has been documented over the past few decades [19, 41], no studies have yet conclusively demonstrated the specific biological importance of Al or Cr at the cellular level. The soil utilized in this study is characterized as optimal for most plant species, with a pH range of 5.9–6.3. Within this range, the risk of Al^3+^ and Cr^3^ toxicity is minimized because their solubility and bioavailability are very low to negligible. Simultaneously, the availability of essential macronutrients—such as phosphorus (P), calcium (Ca), and magnesium (Mg)—is optimized for plant uptake. Under these conditions, Al^3+^ and Cr^3^ primarily exist as insoluble precipitates or are sequestered in stable, non-toxic complexes with organic matter.

Direct soil analysis for Al^3+^ and Cr^3^ was not performed, as the leaf tissue concentration serves as a more reliable indicator of the bioavailable fraction of these metals. Had the soil contained significant mobile fractions of Al or Cr, it would have been reflected in higher leaf accumulation levels. Conversely, our data showed that Al^3+^ and Cr^3^ concentrations in the leaves actually decreased over time as the plants matured. To ensure consumer safety, the concentrations of Al^3+^ and Cr^3^ were monitored to confirm they did not exceed the permissible limits established by the WHO [42]. A critical finding regarding the safety of these nanomaterials is the low residual concentration of Al and Cr within the leaf tissues. The observed temporal decline in elemental levels indicates that the MOFs effectively deliver their stimulatory effects while avoiding the risks associated with heavy metal sequestration. This profile confirms the biocompatibility of Al-MOF and Ag_2_CrO_4_/Al-MOF at the applied concentrations for leafy vegetable production.

This trend is likely attributed to the dilution effect, where the rapid increase in total plant biomass outpaces the rate of metal uptake, thereby lowering the relative concentration of these ions as the plant grows. The baseline levels detected in the control and treatment groups suggest that these trace amounts may have been present in the seeds prior to sowing and subsequently translocated to the foliage. Furthermore, the decrease in detectable mobile ions might be due to several sequestration mechanisms within the plant. These include the binding of Al^3+^ and Cr^3^ to leaf cell walls and xylem vessels [43], compartmentalization within vacuoles [44], or complexation with polysaccharides [45] and organic acids [46] in the leaf tissue. Such interactions effectively immobilize the metals, limiting their bioavailability and transport within the plant system [47].

Several plant growth parameters including phenotype (physical form), shoot height, root length, root to shoot length ratio, number of leaves per plant and leaf area were determined to estimate the effects of different treatments on *Raphanus sativus* growth. Relative to control group, gamma irradiation and/or MOFs treatments significantly enhanced the phenotype, shoot height, root length as well as the leaf area in varying degrees. All Raphanus sativus plants at the starting point, 0 DAS, presented CCI non-significant differences between treatments with the control. Even as the days passed, the CCI did not change significantly within the same treatment across different time points. On the other side, the leaf area varied significantly between treatments at the starting point, 0 DAS. The leaf area also increased significantly within the same treatment across different time points (when comparing 0DAS to 9DAS within the same treatment). The CCI is relatively constant within the same treatment per unit area of the leaf, while the leaf area varied according to the different treatments, with slight variations in the CCI. From this we conclude that the content of chlorophyll increased in the leaf as a whole, and so did the leaf area.

The current study indicates that there is no interaction effect of the treatments—as the statistical results showed—on chlorophyll, stem height, or number of leaves. In other words, the effect observed on these characteristics is due to the main effect of the treatment. However, the synergistic interaction effect between the treatments (a synergistic effect due to the presence of irradiation as a stronger influence) appeared on root length on 0 DAS, 3 DAS and 6 DAS. It also appeared on the root-to-shoot length ratio only on 6 DAS and 9 DAS and on leaf area throughout the whole experiment.

Improved growth features may be due to increased photosynthetic activity and/ or accumulation of suitable osmolytes maintaining plant growth performance [48]. The root activity, formation of new roots, size of root system and consequently the root to shoot length ratio are mainly depend on the nutrients concentration, physical, chemical and biological properties of the root environment. The root to shoot length ratio serves mainly in suppling of the water, nutrients and growth regulators from the root environment. During the reproductive growth, the competition for photosynthates between shoot and roots is the dominant factor limiting root activity and growth [49]. This may illustrate how irradiation resulted in a less root to shoot length ratio through stimulating shoot growth than root growth. These results are consistent with the previous results on oakleaf lettuce seedlings where Pt-NPs foliar exposure showed increased growth of shoots [50]. In addition, Panda et al. [51]. reported increased number of leaves with larger shoot length through foliar NPs-spraying in tomato plants. Moreover, Mubashir et al. [48]. reported increments in plant heights, leaf area and number of leaves per plant in tomato under drought stress through foliar application of nano nutrient solution. Also, Foliar Ag-NPs at 60 ppm, Cu-NPs at 80 ppm, Cu-Ag NPs at 80 ppm and ZnO-NPs at 50 and 100 mg l^− 1^ treatments improved the chili plant growth (increase in plant height and leaf area) [52].

In contrast, the chlorophyll content throughout the study period generally showed non-significant differences compared to the initial levels at 0 DAS—with the exception of Al-MOF-sprayed plants after six days. This stability, despite potential enhancement, may be attributed to a dilution effect resulting from the rapid increase in plant biomass during growth. Mounir et al. [7] suggested that low doses of gamma irradiation can activate the biosynthesis of photosynthetic pigments, whereas high doses typically deplete these parameters. The modulation of the pigment system via gamma irradiation to improve photosynthetic efficiency has been well-documented [6, 41]. Specifically, Tariverdizadeh et al. [53] observed an increase in all photosynthetic pigments following gamma irradiation.

Furthermore, Kim et al. [6] noted that mutagenic treatments using gamma radiation can enhance chlorophyll characteristics, subsequently improving yield components. This dose-dependent response is supported by Hussein [54], who found that barley seeds irradiated with 5 Gy from a ^60^Co source exhibited significantly higher chlorophyll content than the control. Conversely, a higher dose of 20 Gy resulted in a marked reduction in chlorophyll levels compared to the control group. Previous research on foliar exposure of oakleaf lettuce to silver nanoparticles (Ag-NPs) showed only minor changes in chlorophyll concentrations [50]. Conversely, Mubashir et al. [48] reported significant increases in total chlorophyll following the foliar application of nanoparticles in tomato plants under both normal and stressed conditions. Also, TiO_2_ -NPs resulted in increased chlorophyll content and general plant biomass in addition to great enhancement in plant growth and development under heavy metal stress [18]. Furthermore, Emamverdian et al. [55] noted that treated with 150 µM Cr treatment reduced total chlorophyll by 37% in bamboo. The results of the present study suggest that the application of gamma-irradiation, Al-MOF, and/or Ag_2_CrO_4_/Al-MOF may enhance the concentrations of 5-Aminolevulinic acid (ALA). ALA is a crucial growth regulator and a common precursor in the chlorophyll biosynthetic pathway; its upregulation subsequently improves chlorophyll content and photosynthetic function in *Raphanus sativus* [48].

The constructive impacts of low-dose gamma-irradiation, with or without the presence of MOFs, may be ascribed to the elevated synthesis of secondary metabolites and phytochelatins during seedling growth [41]. These improvements may also stem from induced variations in metabolic and defensive signaling pathways [56].

*Raphanus sativus* leaves in the past and until now were utilized for their bioactive compounds’ potential. Goyeneche et al. [57]. reported that ascorbic acid as well as total phenol contents in radish roots were half the value found in the leaves. Furthermore, increased ascorbic acid and phenol contents with irradiation as well as Al-MOF or Ag_2_CrO_4_/Al-MOF further improved the antioxidant system that conveniently improved its nutritional value. L-ascorbic acid is the active form of vitamin C. Like vitamin E, ascorbic acid is a primary defensive nutrient through its function as a free-radical scavenger. Ascorbic acid plays an important role in α-tocopherol regeneration, which has been reported to act as the primary antioxidant [58]. Besides this, ascorbic acid plays many other roles in the antioxidant metabolism. As a maintains a crucial function in many metabolic processes [59]. Mittler [60] reported that ascorbic acid is a non-enzymatic antioxidant compound performing as a reaction substrate within the enzymatic cycle and as an electron donor to reduce the accumulation of reactive oxygen species (ROS). On the other hand, polyphenols have antioxidant capacity higher than carotenoids or vitamins.

Ascorbic acid is a powerful antioxidant that plays a vital role in plant growth and development. It acts as a cofactor for enzymes, regulates cell division and growth, and helps plants withstand various environmental stresses. MOF-nanoparticles can further enhance these benefits, improving plant growth, yield, and stress resilience. It can be observed, in the present study, that on the last day, the high ascorbic acid content was associated with an increase in the number of leaves on the plant, as well as leaf area and shoot height. This suggests that treatments may have affected the ascorbic acid content, which in turn improved the plant’s characteristics.

Plant phenolics constitute a diverse group of secondary metabolites, including coumarins, phenolic acids, stilbenes, flavonoids, condensed and hydrolysable tannins, lignins, and lignans [61]. These compounds serve multifaceted functions in plant physiology, primarily acting as potent antioxidants. The radical-scavenging efficiency of phenolics is determined by their molecular structure, specifically the number and position of hydroxyl (–OH) groups [62].

Phenolic antioxidants mitigate oxidative stress by trapping lipid alkoxyl radicals, thereby inhibiting lipid peroxidation. Furthermore, phenolics contribute to membrane stability by reducing membrane fluidity in a concentration-dependent manner. This physical alteration interferes with the diffusion of free radicals and hinders subsequent peroxidative chain reactions [63]. Previous research has demonstrated that the nutritional value of *Raphanus sativus* leaves significantly exceeds that of the roots [57]. Consequently, the present study focused on evaluating the ascorbic acid and total phenolic content within the shoots of *Raphanus sativus* to assess their antioxidant potential under the experimental treatments.

The present study revealed that total phenol in Raphanus sativus was significantly affected by the application of irradiation and/or MOF nanoparticles, with both increases and decreases observed depending on the type of treatments used. While control plants recorded the least phenol content through the experiment. The current study indicates an interactive effect of the treatments—as shown by the statistical results—revealed on both ascorbic acid content and total phenol. The interaction between the treatments appeared as a synergistic effect resulting from the presence of irradiation and MOF nanoparticles, although irradiation had the greater effect in most cases during the experiment. Notably, Al-MOF was the strongest influence on phenols on 3 DAS, 6 DAS, and 9 DAS when accompanied with irradiation. Similarly, Ag_2_CrO_4_/Al-MOF was the strongest influence on ascorbic acid content when accompanied by irradiation treatment throughout the entire study period. The enhancement of the antioxidant system observed in our study, particularly the increase in phenolic compounds and ascorbic acid, is a common response to various biotic and abiotic stress mitigators. For instance, Emamverdian et al. [64] reported that phytohormones like BAP and ABA boosted antioxidant enzyme activities in bamboo under Cd and Cu stress, paralleling our findings. Similarly, the application of TiO_2_ nanoparticles has been shown to alleviate Cd toxicity by promoting plant growth and physiological function [18]. These studies, along with our own, suggest that both physical (gamma irradiation) and chemical (MOFs, phytohormones, other NPs) elicitors can converge on similar downstream pathways related to antioxidant metabolism and stress tolerance.

Amiri et al. [65] and Aly et al. [66] observed increases in phenolic compounds following gamma-irradiation in common buckwheat and blackberry. Similar increases in phenol content were reported in *Brassica napus*, *Cajanus cajan*, and *Sideroxylon capiri* under UV-B radiation [67, 68]. Gamma-irradiation is known to stimulate the synthesis or activity of enzymes responsible for polyphenolic acid production, particularly phenylalanine ammonia-lyase (PAL), thereby increasing total phenolic concentrations [69, 70]. Furthermore, nanoparticles exert a cumulative effect on antioxidant metabolites, such as ascorbic acid and phenols, as well as various antioxidant enzymes. These compounds mitigate the accumulation of ROS, protecting vital metabolic pathways and enhancing overall plant growth performance [54, 71]. While Jurkow et al. [50] found that Au-NPs, Ag-NPs, and Pt-NPs did not affect the ascorbic acid content in oakleaf lettuce, the application of Ag-NPs and Pt-NPs increased total phenol content by 17% and 15%, respectively, compared to the control.

Molecular markers are highly valuable and very useful. SCoT is a polymorphism marker based on short, conserved sequences adjacent to the ATG start codon in plant genes that were developed by Collard and Mackill [31]. The usage of SCoT markers is deemed more effective than random markers, partly due to their higher annealing temperatures and extended primer distances [31]. El-Khateeb et al. [72]; Riviello-Flores et al. [73] and Talebi et al. [74] discovered that gamma irradiation and nanoparticles led to the development of new genetic markers in mutant individuals while causing the loss of other markers present in the control group; thus, it led to genetic variations in some plant varieties. Hence, it follows that SCoT analysis provides a useful molecular indicator for detecting changes in plants exposed to gamma irradiation and MOFs by detecting DNA polymorphism. Accordingly, the results of similarity and clustering analysis, the Raphanus sativcus genotypes treated with the combination of gamma-irradiation and MOFs were found to be genetically distant from the control. In contrast, the Ag_2_CrO_4_/Al-MOF treatment alone remained closely related to the control group. These results indicate that the synergistic application of gamma-irradiation and the MOF composites induced more significant genetic variations than individual applications. In this study, the gamma-irradiation + Ag_2_CrO_4_/Al-MOF treatment resulted in six unique negative markers, suggesting the emergence of genetic variants and a degree of DNA instability relative to the control and other treatments [38, 75, 76].

The unique SCoT polymorphisms observed in the synergy-treated groups suggest that the combination of gamma irradiation and Ag_2_CrO_4_/Al-MOF induces a degree of genomic remodeling. While these alterations correlate with superior phenotypic outcomes, it remains to be determined whether they represent stable, growth-promoting mutations or a transient adaptive response to physicochemical stress. Future investigations focusing on the M_2_ and M_3_ generations are essential to confirm the transgenerational heritability of these markers. Such studies will clarify whether this synergistic approach can be utilized for the permanent improvement of *Raphanus sativus* germplasm or if it serves primarily as a temporary physiological stimulus.

The SCoT marker analysis revealed a distinct genetic distance between the synergy-treated plants (Gamma + MOF) and the control group, which directly correlates with the observed “extreme” phenotypic values in leaf area and polyphenolic content. This divergence suggests that the combined treatment does not merely “speed up” existing metabolic pathways but may trigger a reconfiguration of the gene expression landscape. The unique markers (polymorphisms) identified in the synergy group likely correspond to genomic regions associated with the phenylpropanoid pathway and developmental regulators. This indicates that the physical and chemical stressors (radiation and MOFs) act as targeted elicitors, pushing the plant toward a new physiological equilibrium that maximizes biomass and antioxidant production.

The observed synergy between low-dose gamma irradiation and foliar-applied MOFs suggests a multi-layered interaction that enhances plant vigor more effectively than either treatment in isolation. We propose three primary mechanisms for this integration. First, gamma irradiation, even at hormetic doses, is known to induce subtle modifications in the leaf cuticle and epidermal cell walls. By slightly altering the polymerization of epicuticular waxes or inducing microscopic structural “loosening” in the cell wall matrix, irradiation may lower the physical barriers to nanoparticle entry. This “radiological priming” likely facilitates a higher flux of MOF crystals through the stomatal pores and into the apoplastic space, ensuring a more robust delivery of Al^3+^ and Cr^3+^ ions. Second, the stimulatory effect of low-dose irradiation is primarily rooted in the generation of controlled levels of reactive oxygen species (ROS). These ROS act as signaling molecules that “wake up” the plant’s endogenous antioxidant defense system (e.g., superoxide dismutase, catalase). When MOFs are subsequently applied, the plant is already in a heightened physiological state; the MOFs then provide the structural or catalytic “fuel”—via their metal nodes and organic linkers—to sustain and amplify this upregulated metabolic state, leading to the significant increases in polyphenolic content observed in our results. Third, while irradiation triggers rapid signaling through physical energy absorption, MOFs provide a slow-release chemical signal. The synergy may be a result of temporal complementarity: the gamma rays provide an immediate “metabolic spark,” while the MOFs’ porous structure ensures the sustained release of ions that support long-term enzymatic activities. Specifically, Al^3+^ and Cr^3+^, when delivered in a controlled MOF framework, may act as co-factors for the very enzymes (such as phenylalanine ammonia-lyase) that were initially activated by the radiation treatment.

## Conclusion

Gamma irradiation is a well-established tool for the improvement of agricultural traits in various plant species, including radish (*Raphanus sativus*). The results of this study demonstrate that a low dose of 10 Gy gamma-irradiation, delivered via a ^60^Co source prior to cultivation, exerts a stimulatory impact on the vegetative characteristics of radish. This effect is significantly enhanced when coupled with the foliar application of MOFs, leading to superior overall growth performance and improved physiological constituents. Some results reflected a synergistic relationship between irradiation and foliar application of MOFs. Such a combination provides a viable pathway for producing high-quality radish with optimized vegetative yield. Plants’ physiological studies are critical when implementing new spraying materials to ensure efficacy or maximize beneficial outcomes. Growth metrics, along with other physiological studies, are crucial to identify potential unique and synergistic effects that are stronger than the components individually.

The findings suggest that the integration of gamma-irradiation and MOF technology can be effectively utilized to induce desirable traits in *Raphanus sativus*. However, further longitudinal studies are warranted to comprehensively assess any potential long-term environmental or biological risks associated with the use of the silver chromate/aluminum organic framework Ag_2_CrO_4_/Al-MOF) to ensure its safety and sustainability in agricultural applications.

## Supplementary Information

Supplementary Material 1.

## Data Availability

I do not have any research data outside the submitted manuscript file.
