# Supplementary material for: Enhanced physiological performance and induced genetic variation in radish (Raphanus sativus L.) under gamma irradiation via silver chromate/aluminum-organic framework application
Source: BMC Plant Biol. 2026 Apr 25;26:754. doi: 10.1186/s12870-026-08715-3 (PMC13112832; doi:10.1186/s12870-026-08715-3)
Supplement: Supplementary file 1 — Supplementary Material 1. [file 12870_2026_8715_MOESM1_ESM.docx]

**Table S1.** Influence of various treatments on the number of leaves per plant in *Raphanus sativus* at 0, 3, 6, and 9 days after spray (DAS).

|  |  | No. of Shoot leaves/ Plant | | |  |
| --- | --- | --- | --- | --- | --- |
| Treatment | | 0DAS | 3DAS | 6DAS | 9DAS |
| Non-irradiated | Control | 3.0 | 3.0 | 3.0 | 3.0 |
|  | Al-MOF | 3.0 | 3.0 | 3.0 | 3.0 |
|  | Ag_2_CrO_4_/Al-MOF | 3.0 | 3.0 | 3.0 | 4.0 |
| Irradiated | Control | 3.0 | 3.0 | 4.0 | 4.0 |
|  | Al-MOF | 4.0 | 4.0 | 4.0 | 4.0 |
|  | Ag_2_CrO_4_/Al-MOF | 3.0 | 4.0 | 4.0 | 4.0 |

Means (n = 3) with standard error (SE = 0) meaning every data point in the dataset of the same treatment is exactly the same as the mean. DAS=Day after spray.


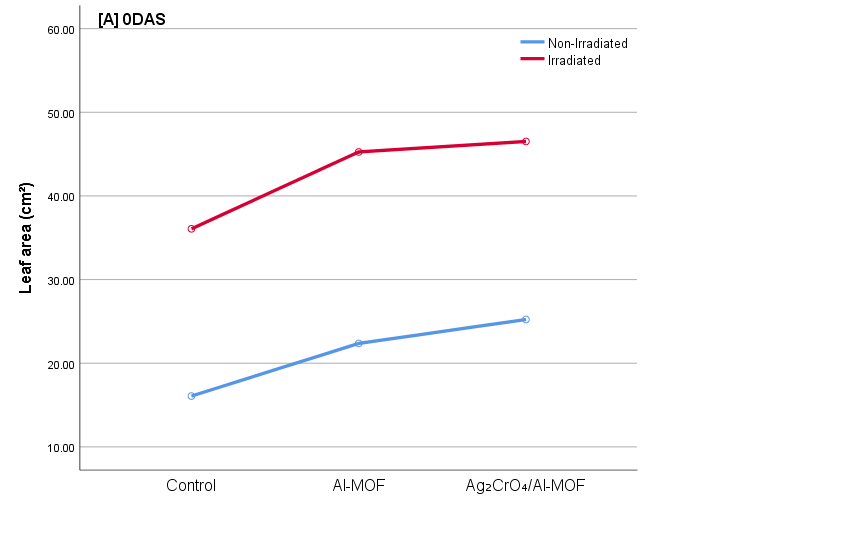

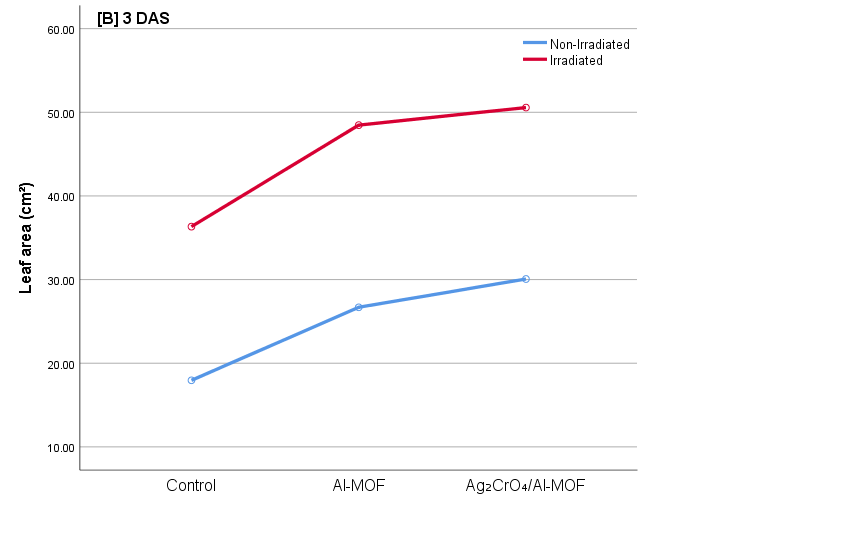


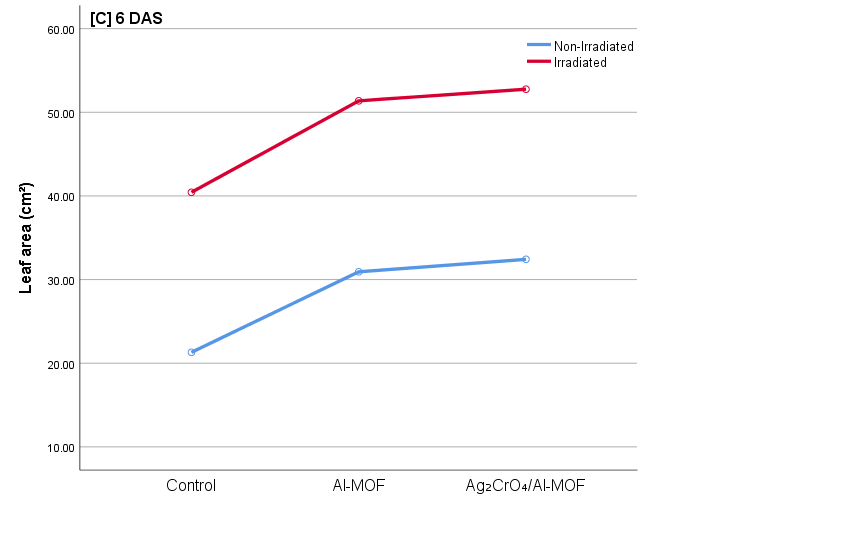

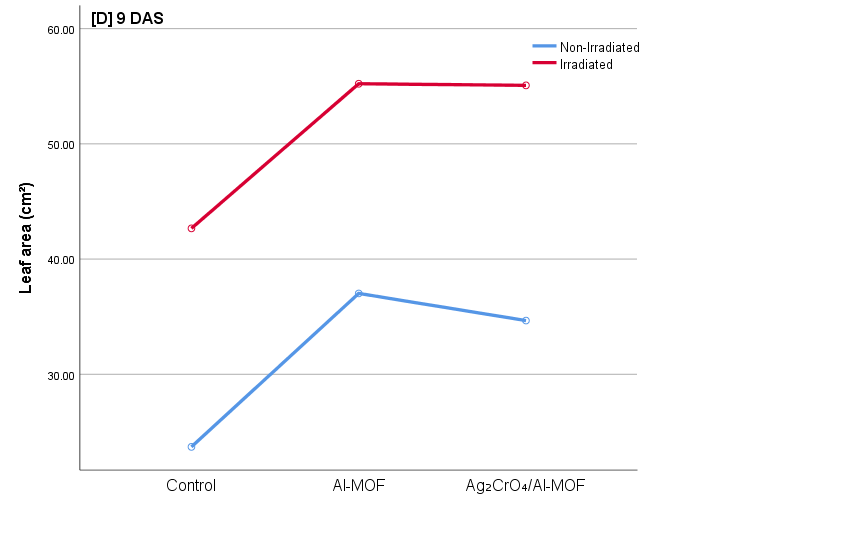


**Figure S1**. Interaction effect between the treatments and its effect on the leaf area across different time points 0 DAS [A], 3 DAS [B], 6 DAS [C] and 9 DAS [D].


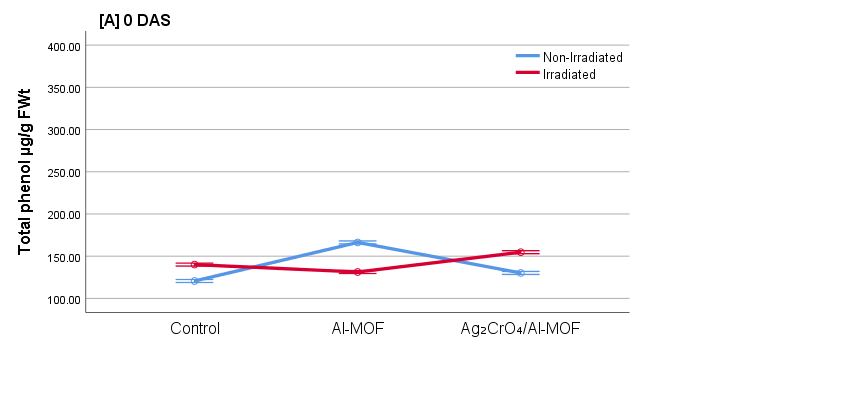

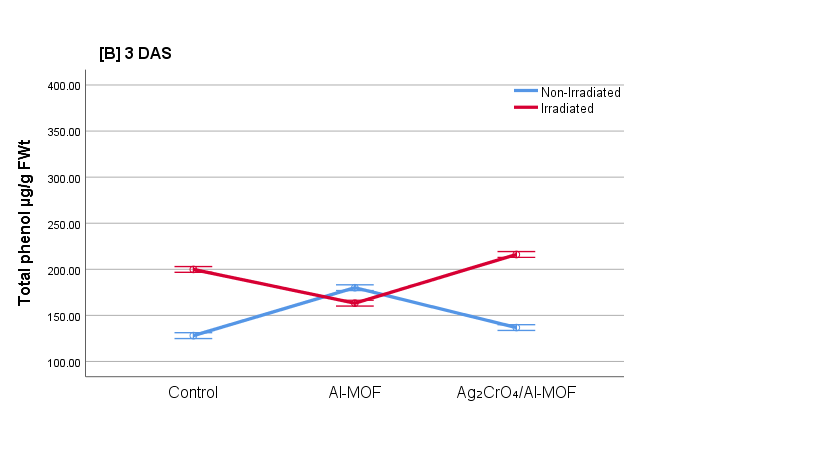


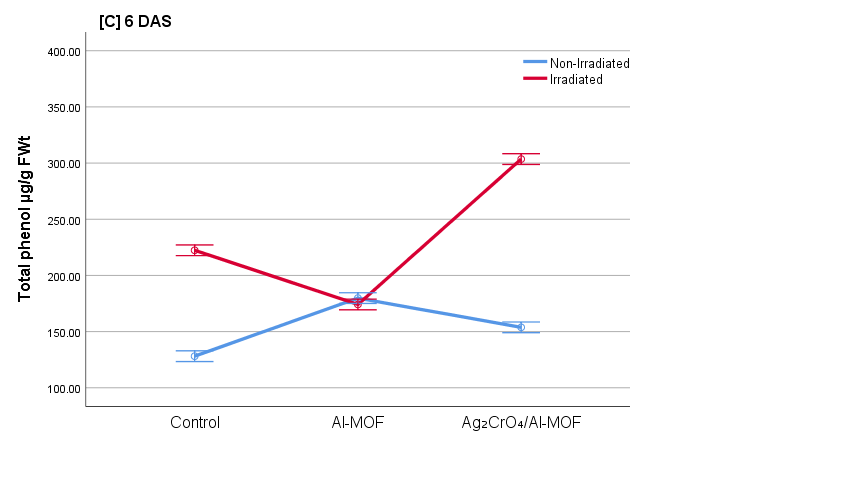

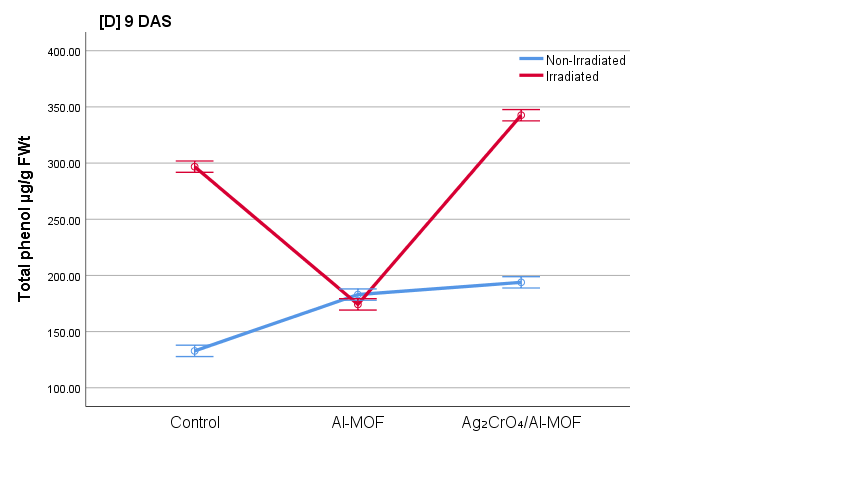


**Figure S2.** Interaction effect between the treatments and its effect on the phenol content across different time points 0 DAS [A], 3 DAS [B], 6 DAS [C] and 9 DAS [D].


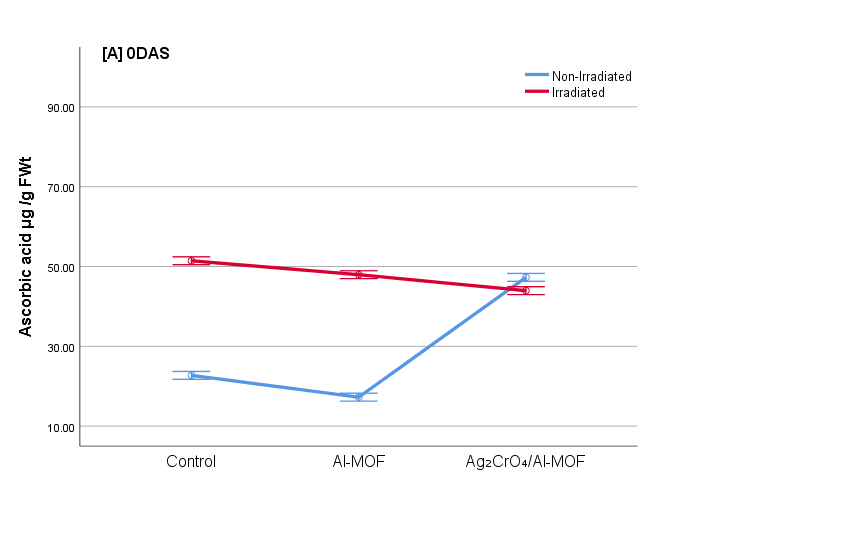

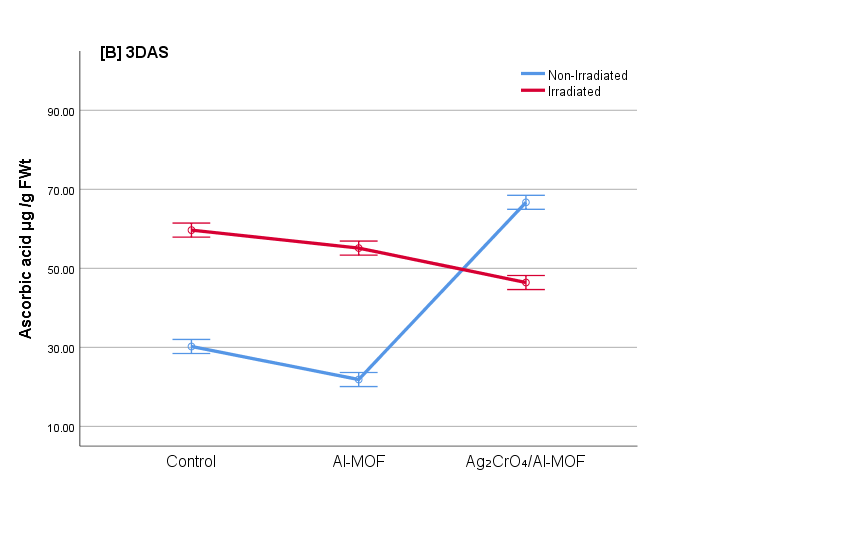


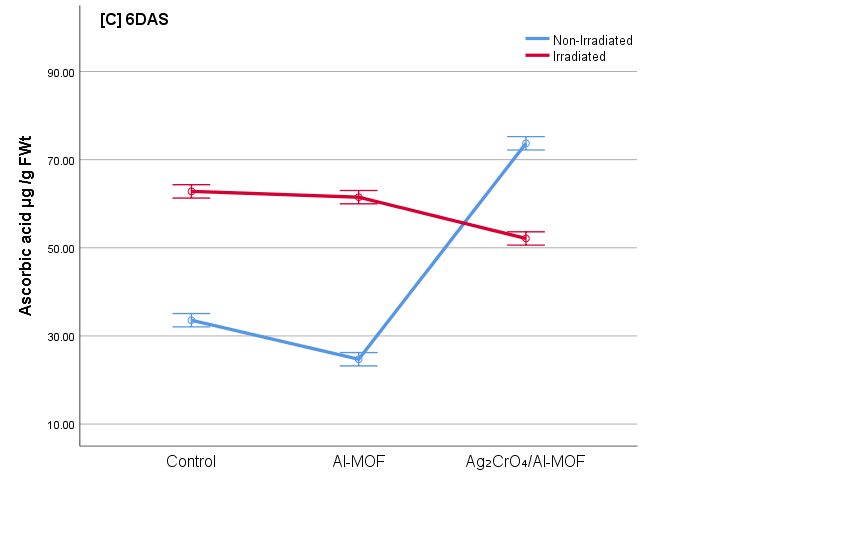

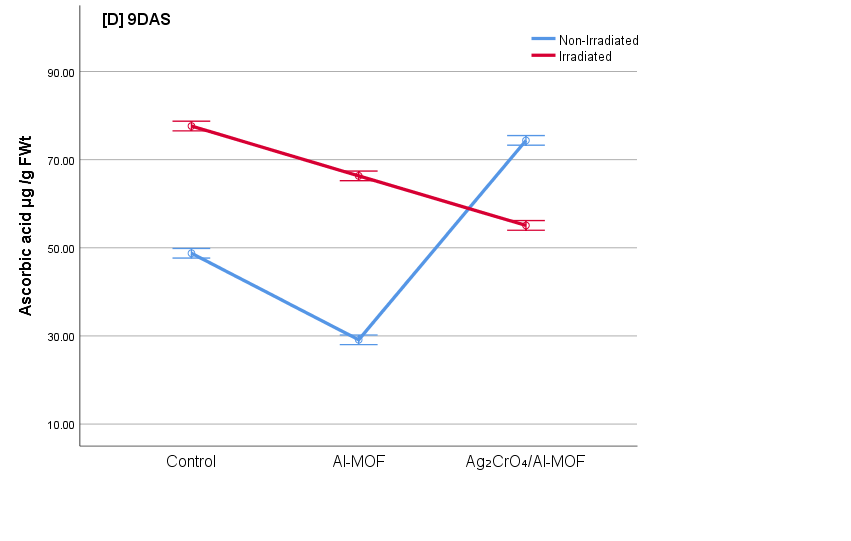


**Figure S3.** Interaction effect between the treatments and its effect on the ascorbic acid content across different time points 0 DAS [A], 3 DAS [B], 6 DAS [C] and 9 DAS [D].

**Supporting information**

SCoTssss-3


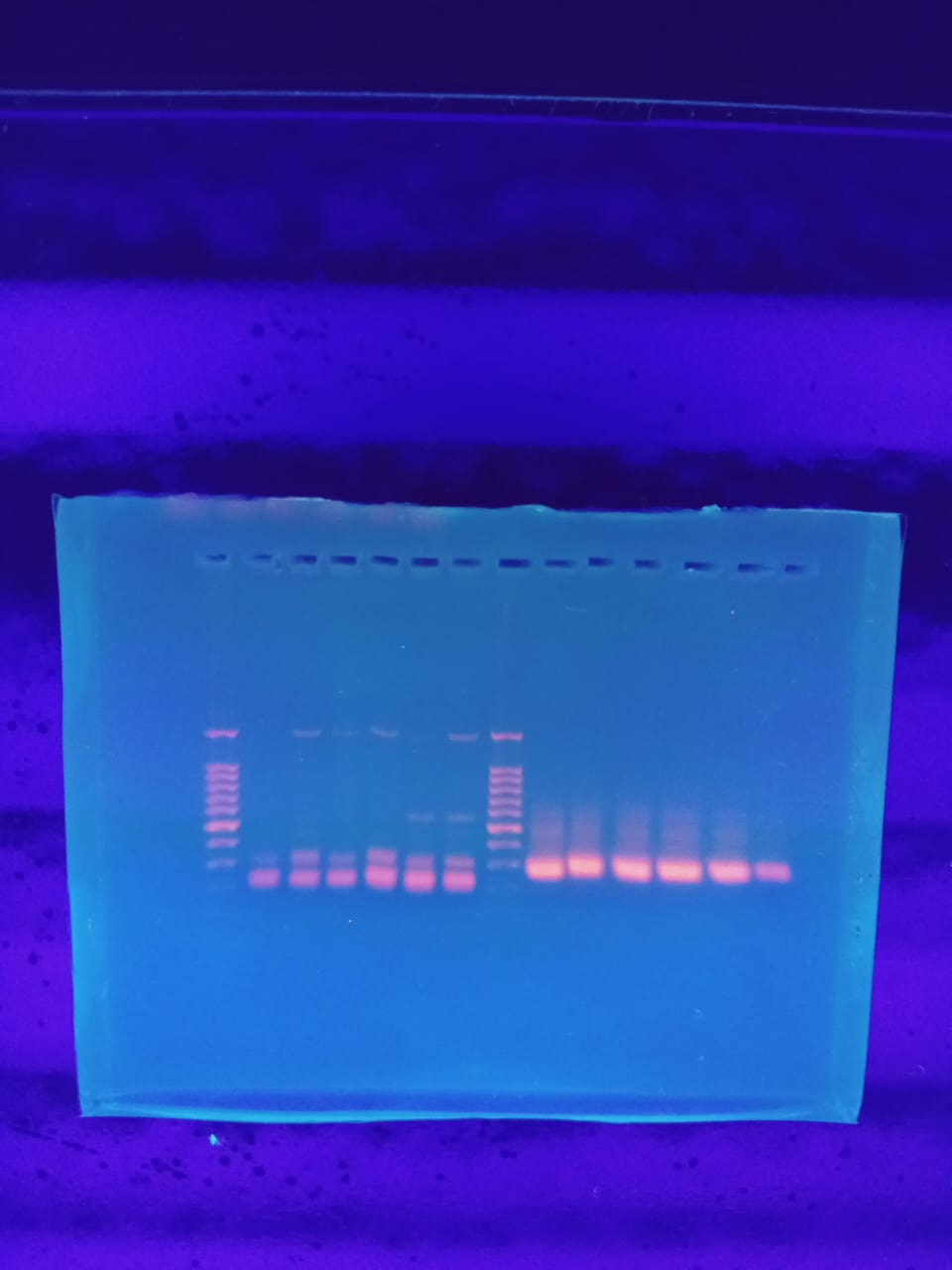


SCoT-4


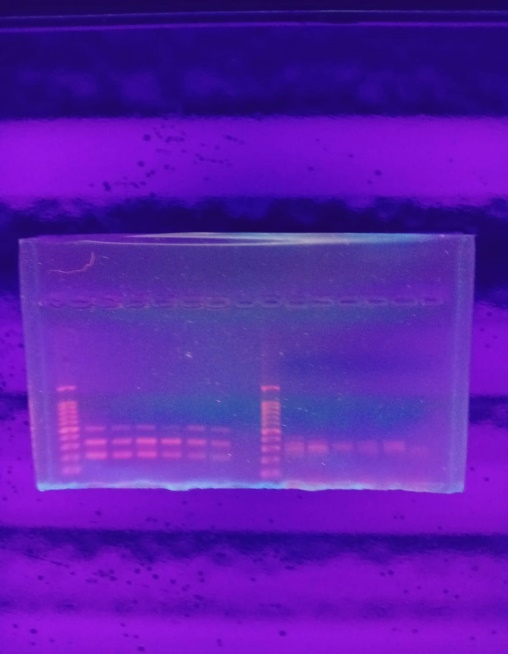


SCoT-5

SCoT-7


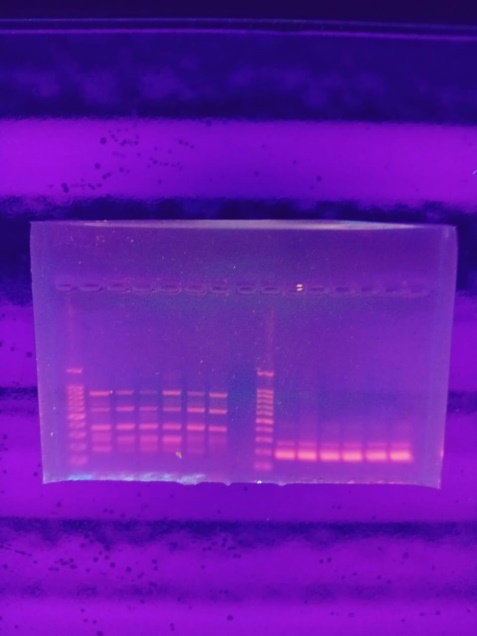


SCoT-1

SCoT-2

SCoT-3

**
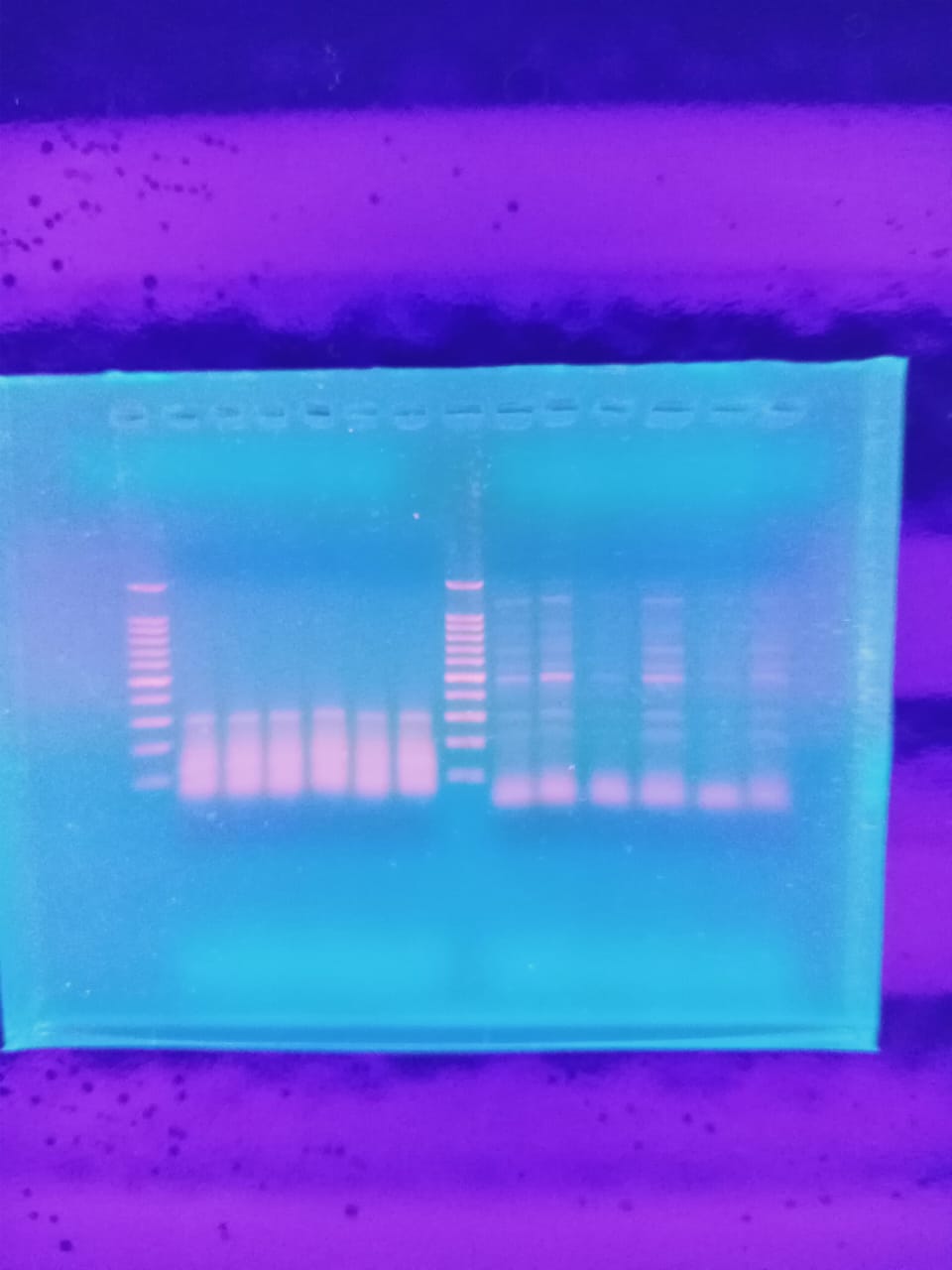
**


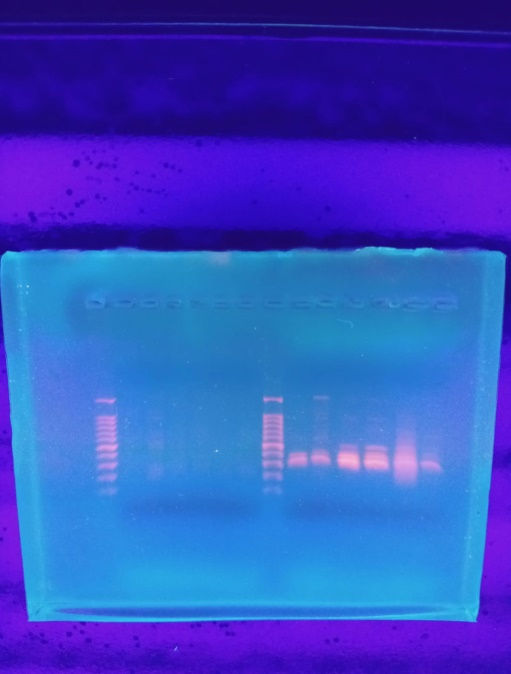


SCoT-9


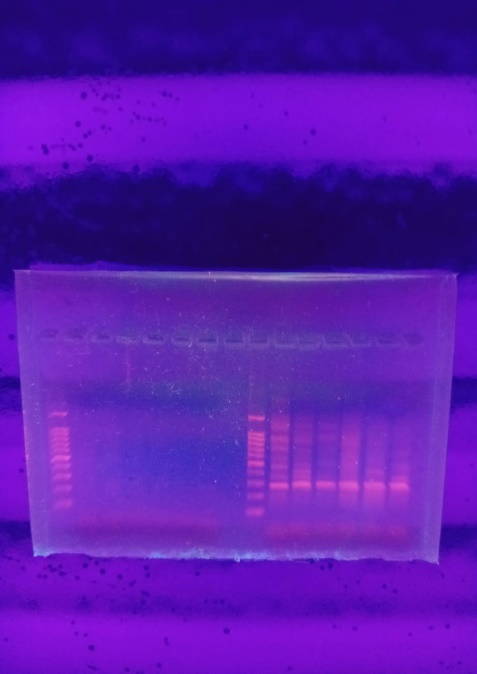


SCoT-10

SCoT-14

SCoT-12

**Figure S4. SCoT results for primer SCoT-1, SCoT-2, SCoT-3, SCoT-4, SCoT-5, SCoT-7, SCoT-9, SCoT-10, SCoT-12, and SCoT-14.**

| **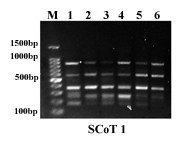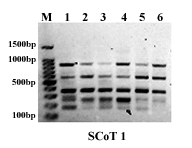** | \| Band  No \| M.W  bp \|  \| \| \| \| \| \| \| --- \| --- \| --- \| --- \| --- \| --- \| --- \| --- \| \| 1 \| 2 \| 3 \| 4 \| 5 \| 6 \| \| 1 \| 830 \| 1 \| 1 \| 1 \| 1 \| 1 \| 1 \| \| 2 \| 580 \| 1 \| 1 \| 1 \| 1 \| 1 \| 1 \| \| 3 \| 345 \| 1 \| 1 \| 1 \| 1 \| 1 \| 1 \| \| 4 \| 275 \| 1 \| 0 \| 0 \| 0 \| 1 \| 1 \| \| 5 \| 255 \| 1 \| 1 \| 1 \| 1 \| 0 \| 0 \| \| 6 \| 185 \| 1 \| 1 \| 1 \| 1 \| 1 \| 0 \| \| Total \| \| 6 \| 5 \| 5 \| 5 \| 5 \| 4 \| |
| --- | --- | --- | --- | --- | --- | --- | --- | --- | --- | --- | --- | --- | --- | --- | --- | --- | --- | --- | --- | --- | --- | --- | --- | --- | --- | --- | --- | --- | --- | --- | --- | --- | --- | --- | --- | --- | --- | --- | --- | --- | --- | --- | --- | --- | --- | --- | --- | --- | --- | --- | --- | --- | --- | --- | --- | --- | --- | --- | --- | --- | --- | --- | --- | --- | --- | --- | --- | --- | --- | --- | --- |

| 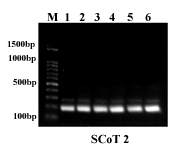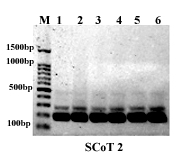 | \| Band  No \| M.W  bp \|  \| \| \| \| \| \| \| --- \| --- \| --- \| --- \| --- \| --- \| --- \| --- \| \| 1 \| 2 \| 3 \| 4 \| 5 \| 6 \| \| 1 \| 385 \| 1 \| 1 \| 1 \| 1 \| 1 \| 1 \| \| 2 \| 185 \| 1 \| 1 \| 1 \| 1 \| 1 \| 1 \| \| Total \| \| 2 \| 2 \| 2 \| 2 \| 2 \| 2 \| |
| --- | --- | --- | --- | --- | --- | --- | --- | --- | --- | --- | --- | --- | --- | --- | --- | --- | --- | --- | --- | --- | --- | --- | --- | --- | --- | --- | --- | --- | --- | --- | --- | --- | --- | --- | --- | --- | --- | --- | --- |

| 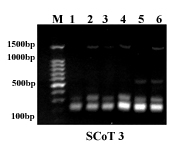 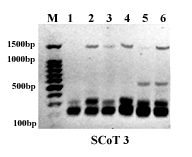 | \| Band  No \| M.W  bp \|  \| \| \| \| \| \| \| --- \| --- \| --- \| --- \| --- \| --- \| --- \| --- \| \| 1 \| 2 \| 3 \| 4 \| 5 \| 6 \| \| 1 \| 1500 \| 0 \| 1 \| 1 \| 1 \| 1 \| 1 \| \| 2 \| 560 \| 0 \| 0 \| 0 \| 0 \| 1 \| 1 \| \| 3 \| 335 \| 1 \| 1 \| 1 \| 1 \| 1 \| 1 \| \| 4 \| 240 \| 1 \| 1 \| 1 \| 1 \| 1 \| 1 \| \| Total \| \| 2 \| 3 \| 3 \| 3 \| 4 \| 4 \| |
| --- | --- | --- | --- | --- | --- | --- | --- | --- | --- | --- | --- | --- | --- | --- | --- | --- | --- | --- | --- | --- | --- | --- | --- | --- | --- | --- | --- | --- | --- | --- | --- | --- | --- | --- | --- | --- | --- | --- | --- | --- | --- | --- | --- | --- | --- | --- | --- | --- | --- | --- | --- | --- | --- | --- | --- |

| 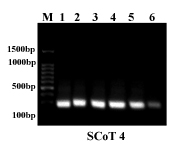 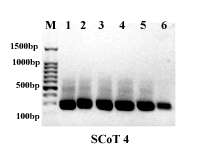 | \| Band  No \| M.W  bp \|  \| \| \| \| \| \| \| --- \| --- \| --- \| --- \| --- \| --- \| --- \| --- \| \| 1 \| 2 \| 3 \| 4 \| 5 \| 6 \| \| 1 \| 520 \| 1 \| 1 \| 1 \| 1 \| 1 \| 0 \| \| 2 \| 325 \| 1 \| 1 \| 1 \| 1 \| 1 \| 1 \| \| 3 \| 300 \| 1 \| 1 \| 1 \| 1 \| 1 \| 1 \| \| Total \| \| 3 \| 3 \| 3 \| 3 \| 3 \| 2 \| |
| --- | --- | --- | --- | --- | --- | --- | --- | --- | --- | --- | --- | --- | --- | --- | --- | --- | --- | --- | --- | --- | --- | --- | --- | --- | --- | --- | --- | --- | --- | --- | --- | --- | --- | --- | --- | --- | --- | --- | --- | --- | --- | --- | --- | --- | --- | --- | --- |

| 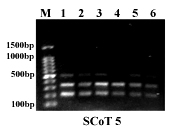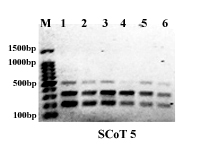 | \| Band  No \| M.W  bp \|  \| \| \| \| \| \| \| --- \| --- \| --- \| --- \| --- \| --- \| --- \| --- \| \| 1 \| 2 \| 3 \| 4 \| 5 \| 6 \| \| 1 \| 500 \| 1 \| 1 \| 1 \| 1 \| 1 \| 1 \| \| 2 \| 320 \| 1 \| 1 \| 1 \| 1 \| 1 \| 1 \| \| 3 \| 200 \| 1 \| 1 \| 1 \| 1 \| 1 \| 1 \| \| Total \| \| 3 \| 3 \| 3 \| 3 \| 3 \| 3 \| |
| --- | --- | --- | --- | --- | --- | --- | --- | --- | --- | --- | --- | --- | --- | --- | --- | --- | --- | --- | --- | --- | --- | --- | --- | --- | --- | --- | --- | --- | --- | --- | --- | --- | --- | --- | --- | --- | --- | --- | --- | --- | --- | --- | --- | --- | --- | --- | --- |

| **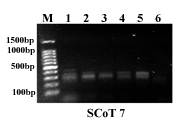** **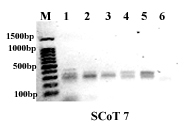** | \| Band  No \| M.W  bp \|  \| \| \| \| \| \| \| --- \| --- \| --- \| --- \| --- \| --- \| --- \| --- \| \| 1 \| 2 \| 3 \| 4 \| 5 \| 6 \| \| 1 \| 435 \| 1 \| 0 \| 0 \| 0 \| 0 \| 0 \| \| 2 \| 365 \| 1 \| 1 \| 1 \| 1 \| 1 \| 0 \| \| 3 \| 275 \| 1 \| 1 \| 1 \| 1 \| 1 \| 1 \| \| Total \| \| 3 \| 2 \| 2 \| 2 \| 2 \| 1 \| |
| --- | --- | --- | --- | --- | --- | --- | --- | --- | --- | --- | --- | --- | --- | --- | --- | --- | --- | --- | --- | --- | --- | --- | --- | --- | --- | --- | --- | --- | --- | --- | --- | --- | --- | --- | --- | --- | --- | --- | --- | --- | --- | --- | --- | --- | --- | --- | --- |

| **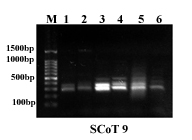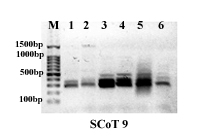** | \| Band  No \| M.W  bp \|  \| \| \| \| \| \| \| --- \| --- \| --- \| --- \| --- \| --- \| --- \| --- \| \| 1 \| 2 \| 3 \| 4 \| 5 \| 6 \| \| 1 \| 1865 \| 0 \| 1 \| 0 \| 0 \| 0 \| 0 \| \| 2 \| 520 \| 0 \| 0 \| 1 \| 1 \| 1 \| 1 \| \| 3 \| 375 \| 1 \| 1 \| 1 \| 1 \| 1 \| 1 \| \| 4 \| 300 \| 1 \| 1 \| 1 \| 1 \| 1 \| 1 \| \| Total \| \| 2 \| 3 \| 3 \| 3 \| 3 \| 3 \| |
| --- | --- | --- | --- | --- | --- | --- | --- | --- | --- | --- | --- | --- | --- | --- | --- | --- | --- | --- | --- | --- | --- | --- | --- | --- | --- | --- | --- | --- | --- | --- | --- | --- | --- | --- | --- | --- | --- | --- | --- | --- | --- | --- | --- | --- | --- | --- | --- | --- | --- | --- | --- | --- | --- | --- | --- |

| **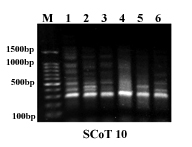** **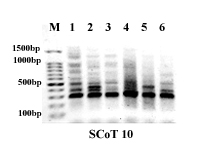** | \| Band  No \| M.W  bp \|  \| \| \| \| \| \| \| --- \| --- \| --- \| --- \| --- \| --- \| --- \| --- \| \| 1 \| 2 \| 3 \| 4 \| 5 \| 6 \| \| 1 \| 1875 \| 1 \| 1 \| 1 \| 1 \| 0 \| 0 \| \| 2 \| 1265 \| 1 \| 1 \| 1 \| 1 \| 1 \| 1 \| \| 3 \| 945 \| 1 \| 1 \| 1 \| 1 \| 1 \| 0 \| \| 4 \| 730 \| 1 \| 1 \| 1 \| 1 \| 0 \| 1 \| \| 5 \| 615 \| 1 \| 1 \| 1 \| 1 \| 1 \| 1 \| \| 6 \| 500 \| 1 \| 1 \| 1 \| 1 \| 1 \| 0 \| \| 7 \| 460 \| 0 \| 1 \| 0 \| 0 \| 1 \| 0 \| \| 8 \| 400 \| 1 \| 1 \| 1 \| 1 \| 1 \| 0 \| \| 9 \| 315 \| 1 \| 1 \| 1 \| 1 \| 1 \| 1 \| \| Total \| \| 8 \| 9 \| 8 \| 8 \| 7 \| 4 \| |
| --- | --- | --- | --- | --- | --- | --- | --- | --- | --- | --- | --- | --- | --- | --- | --- | --- | --- | --- | --- | --- | --- | --- | --- | --- | --- | --- | --- | --- | --- | --- | --- | --- | --- | --- | --- | --- | --- | --- | --- | --- | --- | --- | --- | --- | --- | --- | --- | --- | --- | --- | --- | --- | --- | --- | --- | --- | --- | --- | --- | --- | --- | --- | --- | --- | --- | --- | --- | --- | --- | --- | --- | --- | --- | --- | --- | --- | --- | --- | --- | --- | --- | --- | --- | --- | --- | --- | --- | --- | --- | --- | --- | --- | --- | --- | --- |

| **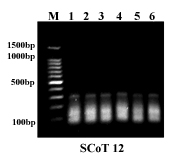** **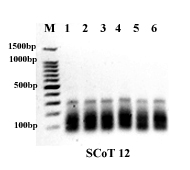** | \| Band  No \| M.W  bp \|  \| \| \| \| \| \| \| --- \| --- \| --- \| --- \| --- \| --- \| --- \| --- \| \| 1 \| 2 \| 3 \| 4 \| 5 \| 6 \| \| 1 \| 300 \| 1 \| 1 \| 1 \| 1 \| 1 \| 1 \| \| 2 \| 175 \| 1 \| 1 \| 1 \| 1 \| 1 \| 1 \| \| 3 \| 160 \| 1 \| 1 \| 1 \| 1 \| 1 \| 1 \| \| 4 \| 86 \| 1 \| 1 \| 1 \| 1 \| 1 \| 1 \| \| Total \| \| 4 \| 4 \| 4 \| 4 \| 4 \| 4 \| |
| --- | --- | --- | --- | --- | --- | --- | --- | --- | --- | --- | --- | --- | --- | --- | --- | --- | --- | --- | --- | --- | --- | --- | --- | --- | --- | --- | --- | --- | --- | --- | --- | --- | --- | --- | --- | --- | --- | --- | --- | --- | --- | --- | --- | --- | --- | --- | --- | --- | --- | --- | --- | --- | --- | --- | --- |

| **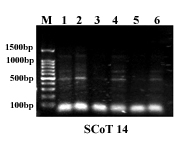** **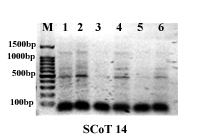** | \| Band  No \| M.W  bp \|  \| \| \| \| \| \| \| --- \| --- \| --- \| --- \| --- \| --- \| --- \| --- \| \| 1 \| 2 \| 3 \| 4 \| 5 \| 6 \| \| 1 \| 1365 \| 1 \| 1 \| 0 \| 1 \| 0 \| 1 \| \| 2 \| 765 \| 0 \| 1 \| 0 \| 1 \| 0 \| 0 \| \| 3 \| 725 \| 1 \| 1 \| 1 \| 1 \| 1 \| 1 \| \| 4 \| 500 \| 1 \| 1 \| 1 \| 1 \| 1 \| 1 \| \| 5 \| 320 \| 1 \| 1 \| 0 \| 1 \| 1 \| 1 \| \| Total \| \| 4 \| 5 \| 2 \| 5 \| 3 \| 4 \| |
| --- | --- | --- | --- | --- | --- | --- | --- | --- | --- | --- | --- | --- | --- | --- | --- | --- | --- | --- | --- | --- | --- | --- | --- | --- | --- | --- | --- | --- | --- | --- | --- | --- | --- | --- | --- | --- | --- | --- | --- | --- | --- | --- | --- | --- | --- | --- | --- | --- | --- | --- | --- | --- | --- | --- | --- | --- | --- | --- | --- | --- | --- | --- | --- |
